# The effects of polysaccharides from *Inonotus obliquus*, artemisinin, and dihydroartemisinin on the reproductive system of male mice infected with Neospora caninum

**DOI:** 10.3389/fvets.2026.1740054

**Published:** 2026-02-20

**Authors:** Jianhao Zhao, Fanglin Zhao, Yang Liang, Zhenyu Wang, Pengfei Min, Yixuan Jin, Lu Li, Zhen Ma, Yang Wang, Xin Zhang, Siyuan Han, Lijun Jia

**Affiliations:** Engineering Research Center of North-East Cold Region Beef Cattle Science and Technology Innovation, Ministry of Education, Yanbian University, Yanji, China

**Keywords:** artemisinin, dihydroartemisinin, *Inonotus obliquus* polysaccharides, male reproduction, Neospora caninum, oxidative stress

## Abstract

The present study investigated the protective effects of *Inonotus obliquus* polysaccharides (IOPs), artemisinin, and dihydroartemisinin (DHA) on Neospora caninum-induced damage of the reproductive system in male BALB/c mice. This study conducted in vivo resistance experiments against Neosporidium using three drugs: polysaccharides from Betula brownii, artemisinin, and dihydroartemisinin. On the basis of establishing a male mouse animal model of new sporidiosis, after gavage administration, the reproductive organ index of male mice was measured at 7d, 14d, 21d, 35d, and 42d, respectively. HE staining and transmission electron microscopy were used to observe the pathological changes of testicular and epididymal tissues. The improved Pap staining method was used to analyze sperm quality, flow cytometry was used to detect apoptosis of spermatogenic cells, MDA and ACP activities were measured, and ELISA was used to detect immunoglobulin IgG1, IgG2a, IgE, and cytokine IFN-γ, IL-4, IL-6, TNF-α. The qPCR method was used to detect the expression of apoptotic genes Bax, Bcl-2, Caspase-3, P53, as well as sperm related genes C-kit, Plzf, Sycp3, Stra8, Dnajb13, Mrto4, and Ipo11, as well as the levels of NO and AsAb. The results showed that IOPs and DHA exhibited significant anti-neosporal activity. Compared to infected mice, IOPs-treated mice showed significantly increased sperm density (*p* < 0.05) and sperm motility (*p* < 0.05), while DHA-treated mice exhibited a remarkably reduced sperm deformity rate (*p* < 0.05). Compared with the model group, the sperm motility in the ART-treated mice was significantl upregulated (*p* < 0.01). Histopathological analysis revealed that all three treatments ameliorated testicular and epididymal tissue damage, reduced mitochondrial vacuolization, and improved organ indices. Biochemical assays showed a reduced level of malondialdehyde and a high level of acid phosphatase activity in the testicular tissue of treated mice. Immunological assays confirmed decreased levels of immunoglobulin G1 (IgG1), IgG2a, interleukin (IL)-4, IL-6, tumor necrosis-α factor nitric oxide, and anti-sperm antibodies in the treatment groups. Gene expression analysis indicated that IOPs significantly downregulated the expression of caspase-3, p53, and Dnajb13 and upregulated expression of SYCP3 and Stra8. There was no significant difference in the ART group (*p* < 0.05). DHA markedly reduced Dnajb13 expression but enhanced SYCP3, Stra8, and Ipo11 expression. These findings suggest that ART has no obvious therapeutic effect on male mice infected with Neospora caninum, IOPs and DHA can effectively mitigate N. caninum induced Spermatogenesis block, spermatozoon maturation impairment, and spermatozoon structural defects in male mice antioxidant, immunomodulatory, and antiapoptotic effects and could be considered promising candidates for anti-neosporal therapy.

## 1 Introduction

Neosporosis is a disease caused by the protozoan parasite Neospora caninum that leads to abortion, stillbirth, and other reproductive disorders in pregnant animals. It occurs as a severe infectious disease in cattle and dogs worldwide (1–3). Currently, there are still many deficiencies in the research on the prevention and treatment of neosporiasis, and there is no effective commercial vaccine or specific drug for the prevention and control of neosporiasis. Moreover, chemical drugs induce several side effects in the infected host and therefore cannot be administered for a longer duration (4). Traditional Chinese medicine (TCM) is being increasingly investigated as an alternative method to prevent and treat parasitic diseases because of its low cost, high efficacy, and low toxicity. Therefore, it is critical to find new TCM-based drugs that can effectively inhibit neosporosis.

*Inonotus obliquus* (Chaga) is a medicinal fungus growing in cold habitats and belongs to the family Polyporaceae, the order Agaricales, the subphylum Basidiomycetes, the class Lycoperdales, and the genus Phellinus (5). *I. obliquus* polysaccharides (IOPs) are considered one of the main bioactive components of *I. obliquus* extracts and exhibit antitumor, antioxidant, antiviral, hypoglycemic, and hypolipidemic effects (5, 6).

Artemisinin (ART), an ancient Chinese herbal drug, has a novel chemical structure and unique antimalarial activity. ART is promptly absorbed, widely distributed, and rapidly metabolized in the body, and it can penetrate the blood–brain barrier. ART is particularly suitable for treating cerebral malaria and severe malaria (7, 8).

Dihydroartemisinin (DHA), an active metabolite of ART and its derivatives, is also an effective clinical drug widely used to treat malaria (9). Compared to ART, DHA has a better metabolic profile, higher bioavailability, broader spectrum activity, lower toxicity and side effects, and lower cost. Moreover, DHA can disrupt the surface membrane and mitochondrial functions of Plasmodium (10).

Spermatogenesis is a germ cell-specific process in male animals that plays a critical role in reproduction. While conserved across species, the detailed cytological steps can vary. For instance, murine spermatogenesis is commonly described in 16 steps across 4 main morphological phases: the Golgi phase, cap phase, acrosomal phase, and maturation phase (11). In mammals, the overarching events of spermatogenesis include acrosome biogenesis, sperm head shaping, and sperm tail (flagellum) development (12). This process is a highly complex and dynamic sequence, encompassing three fundamental stages: spermatogonial proliferation and differentiation, meiosis, and spermiogenesis. Each stage is tightly regulated by specific molecular factors. The self-renewal and maintenance of spermatogonial stem cells are governed by factors such as the glial cell line-derived neurotrophic factor (Gdnf), promyelocytic leukemia zinc finger protein (Plzf), and B-cell lymphoma 6 protein (Bcl6). The initiation of meiosis is critically dependent on genes like Stra8 (stimulated by retinoic acid gene 8). Furthermore, the proper formation of the acrosome—a crucial structure for fertilization—involves the functions of proteins like HRB (human immunodeficiency virus type-1 Rev-binding protein), GOPC (Golgi-associated PDZ and coiled-coil motif-containing protein), and VPS54 (vacuolar protein sorting-associated protein 54). This coordinated sequence of cellular differentiation and morphological transformation ensures the continuous production of functional sperm (13–17).

*In vitro* experiments demonstrated that *Inonotus obliquus* polysaccharides, artemisinin, and dihydroartemisinin, respectively, exhibited inhibitory effects against Neospora caninum within their safe concentration ranges (18). At present, research on the prevention and treatment of neosporosis remains insufficient, with no effective commercial vaccines or specific drugs available for its control. Therefore, this study aims to investigate the therapeutic potential of IOPs, ART, and DHA in mitigating reproductive system damage in mice infected with Neospora caninum, thereby providing a foundation for the prevention and control of neosporosis.

## 2 Materials and methods

### 2.1 Chemicals and reagents

The polysaccharides from *Inonotus obliquus* were extracted using reagents such as petroleum ether, 70% methanol, anhydrous ethanol, ether, and acetone, and stored in our laboratory (polysaccharide content: 84%). ART and DHA (purity: 98%) were purchased from Shanghai YuanYe Biotechnology Co., Ltd. High-glucose Dulbecco’s modified Eagle’s medium (DMEM) medium and dimethyl sulfoxide were obtained from Sigma. Fetal bovine serum (FBS) was supplied by Tianjin Haoyang Biological Products Technology Co., Ltd. Phosphate-buffered saline (PBS) powder, trypsin, and modified a PAP staining kit (EA50) were provided by Solarbio. Penicillin and streptomycin were obtained from Nanjing Novozan Biotechnology Co., Ltd. Absolute ethanol, methanol, glutaraldehyde (domestic analytical and pure grade), and Annexin V apoptosis detection kit were purchased from Sangon Biotech (Shanghai) Co., Ltd. The malondialdehyde (MDA) assay and acid phosphatase (ACP) assay kits were procured from Nanjing Jiancheng Bioengineering Institute (Nanjing, China). Mouse immunoglobulin G1 (IgG1), IgG2a, IgE, interferon-*γ* (IFN-γ), interleukin (IL)-4, and nitric oxide (NO) ELISA kits were supplied by Shanghai Langton Biotechnology Co., Ltd. Mouse IL-6 and tumor necrosis factor-*α* (TNF-α) ELISA kits were provided by Jiangsu enzyme immunoassay Industrial Co., Ltd. The RNA extraction kit was obtained from Promega (Beijing) Biotechnology Co., Ltd. Genomic cDNA first strand synthesis premix reagent and the FastKing one-step reverse transcription-fluorescence quantification kit (SYBR Green) were obtained from Tiangen Biotech (Beijing) Co., Ltd. The mouse anti-sperm antibody (AsAb) ELISA kit was provided by Jingmei Biotechnology Co., Ltd.

### 2.2 Culture and purification of Neospora caninum

A bovine strain of N. caninum was isolated from bovine fetal brain tissue and cultured in the Laboratory of Preventive Veterinary Medicine, Yanbian University, Yanji, China. Vero cells were also maintained in the Laboratory of Preventive Veterinary Medicine, Yanbian University. N. caninum was propagated in Vero cells, and the cells were then cultured in DMEM. Subsequently, 8% heat-inactivated FBS, 100 mg/mL penicillin, and 10 mg/mL streptomycin were added to the cells, and the cells were then incubated at 37 °C in a 5% CO2 environment. Infected Vero cells were washed with ice-cold PBS to purify tachyzoites. Subsequently, first, filter four times using a 27-gage needle, and then filter through a 5-micron filter. The number of parasite cells was determined using a blood cell counting plate.

### 2.3 Animal experiments

**Figure 1:**
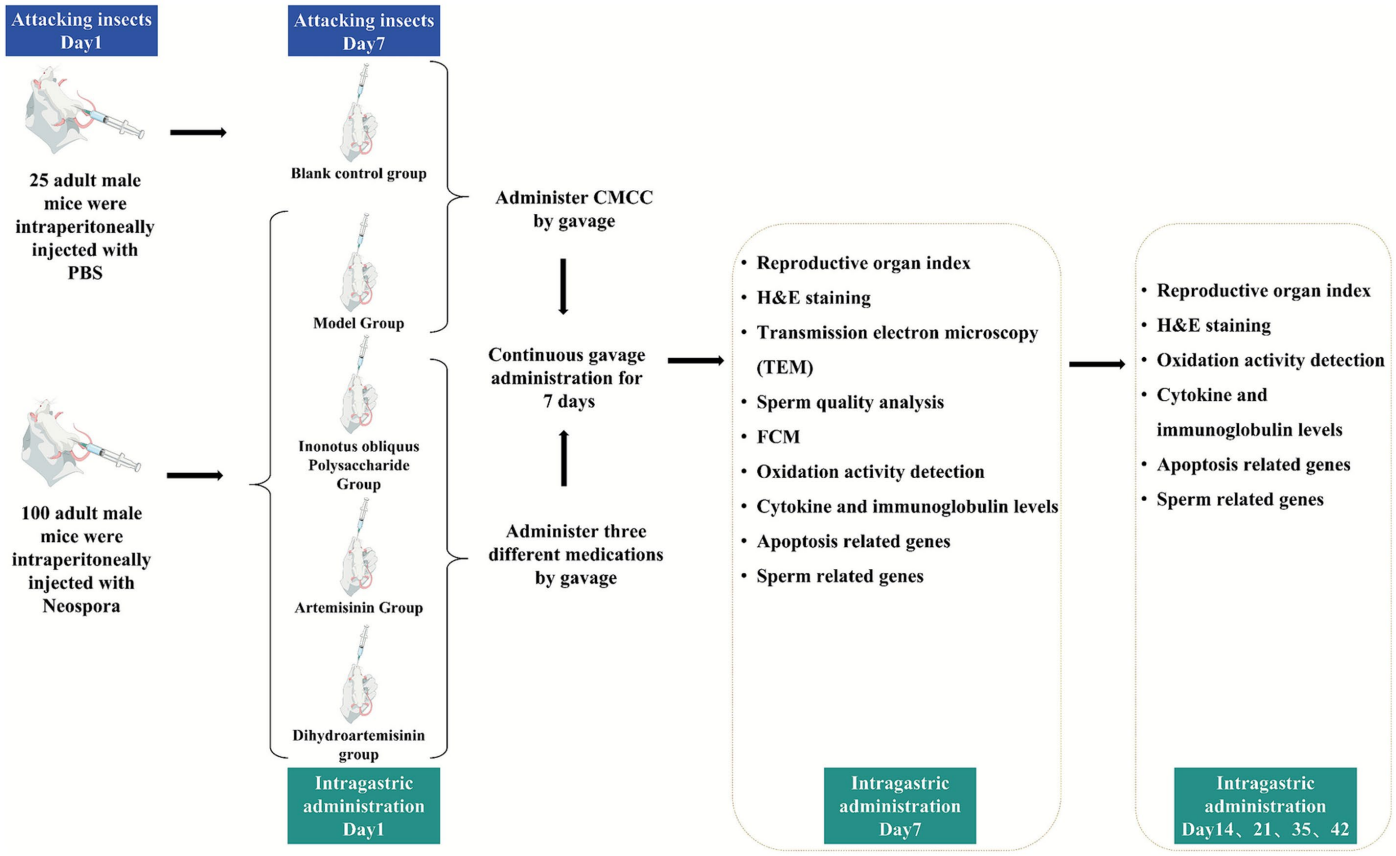
Flowchart of animal experiment in this study.

Male BALB/c mice (age: 8 weeks; weight: approximately 20 g) were housed under controlled temperature (25 ± 1 °C) and humidity (50% ± 5%) with free access to food and water. After 1 week of adaptation to housing conditions, all animals were used for the experiment. The study protocol strictly followed the recommendations provided in the Guide for the Care and Use of Laboratory Animals, Yanbian University. A total of 125 mice were randomly selected, and all mice were randomly assigned to five groups: blank control group, infected model group, IOPs-treated group, ART-treated group, and DHA-treated group, with 25 mice in each group. The male BALB/c mouse model of N. caninum infection was established according to the method of Hang Li (19). The blank control group received an intraperitoneal injection of 0.1 mL of PBS solution, while the remaining four groups were injected with 0.1 mL of a Neospora caninum/PBS suspension (with a Neospora caninum concentration of 1 × 10^6^/mL). Starting from the 7th day after worm infection, the drug was administered by gavage, which was considered as the first day of administration. The dosage was 0.1 mL/10 g, and the drug was continuously administered by gavage for 7 days. The dosage for each group was 200 mg/kg, and the blank control group and model group were given normal saline. Five male mice in each group were sacrificed in batches on the 7th, 14th, 21st, 35th, and 42nd days after treatment administration (19). In accordance with the 3R principles and under a protocol approved by the local Ethical Review Board, mice were euthanized by CO2 asphyxiation using a precision flow meter to ensure a gradual displacement of chamber air. This was immediately followed by cervical dislocation to ensure death prior to tissue collection. After sacrificing the mice, the brains, livers, spleens, testes, and epididymides were dissected and collected for subsequent experiments. Figure 1 is a brief flowchart of the animal experiment part in this study.

### 2.4 Sperm quality analysis and determination of the reproductive organ index

The testes and epididymides were dissected and removed. Subsequently, the adipose tissue and connective tissue were removed from these organs, the organs were weighed, and the reproductive organ index was calculated. The testes or epididymides indices were calculated as the weight of testis or epididymis tissue of each male mice/weight of each male mice (mg/g). Take 0.5 mL of 0.9% saline and place it in a 37 °C water bath. Separate the intact epididymis, place one side of the mouse’s epididymis in preheated physiological saline, and cut it into small pieces. Use a pipette to repeatedly blow and mix the tissue suspension gently for 15–20 times. Place the mixed tissue suspension in a 37 °C constant temperature water bath and wait for the sperm to swim out naturally. Let it sit quietly for 10 min. After preparing the sperm suspension, first observe the sperm motility using a microscope (with a sperm count greater than 200 and at least 5 fields observed). Then, calculate the sperm density using a hemocytometer. Finally, observe the sperm malformation rate through the modified Papanicolaou staining method. The evaluation is conducted within 30 min after sample preparation at 37 °C (20).

### 2.5 Pathological examination

On the 7th, 14th, 21st, 35th, and 42nd days after administration, testicular and epididymal tissues were collected from mice in each group. The tissues were immersed in 10% formalin solution, followed by preparation of paraffin sections (with a thickness of 5 μm) (21), and finally stained with hematoxylin and eosin (H&E).

After the mice were sampled, the tissue blocks were fixed in 4% glutaraldehyde and stored at 4 °C for 1 week. After 1 week, remove it and rinse it three times with PBS buffer for 30 min each time. Fix 1% osmium acid in a refrigerator at 4 °C for 2 h. After fixation, rinse with distilled water 3 times for 15 min each time. Dehydrate using gradient alcohol, acetone, and epichlorohydrin, then pre-soak with a mixture of epichlorohydrin and embedding agent for 1 h, and finally soak overnight with embedding agent. Aggregation is carried out through gradient temperature and ultra-thin slicing is performed. Stain with uranyl acetate and lead citrate, and observe and record under transmission electron microscopy. The transmission electron microscope is sourced from Shanghai Zhujin Analytical Instrument Co., Ltd.

### 2.6 Flow cytometry (FCM) analysis

An Annexin V-FITC/propidium iodide (PI) kit was used to determine cell apoptosis by FCM analysis. Mice testicular tissue was fragmented, and the tissue fragments were filtered through a 300-mesh filter cloth. The obtained cells were resuspended in 1 × binding buffer, and the cell density was adjusted to the appropriate value. Next, Annexin V-FITC was added to the cells, mixed well, and incubated in the dark at room temperature for 10 min. The cells were then washed with 1 × binding buffer and centrifuged, and the supernatant was discarded. The cell layer pellet was resuspended in 1 × binding buffer, 10 μL PI was added, and the cell suspension was immediately placed in the flow cytometer for apoptosis detection. All the experimental steps above were conducted in accordance with the instructions provided in the reagent kit manual.

### 2.7 MDA level and ACP activity determination

Briefly, 0.1 g of the testicular tissue sample was homogenized in 0.9 mL of ice-cold normal saline. The homogenate was centrifuged at 10000 g and 4 °C for 10 min, and the resulting supernatant was used for determining the MDA level and ACP activity. These assays were conducted using the substrate provided by Nanjing Jiancheng Bioengineering Institute in accordance with the manufacturer’s protocol. The optical density was measured using a microplate reader (Bio-Rad), and the MDA and ACP levels were then calculated. All the experimental steps above were conducted in accordance with the instructions provided in the reagent kit manual.

### 2.8 Immunoglobulin subclass and cytokine assays

Blood samples were collected from mice eyeballs on 0, 7, 14, 21, 35, and 42 days after treatment administration, and serum was obtained (centrifugation at 1500 g for 15 min at 4 °C). Serum levels of the immunoglobulin subclasses IgG1, IgG2a, and IgE and the cytokines IFN-*γ*, IL-4, IL-6, and TNF-*α* were detected by following the procedures mentioned in the assay kit instructions. All the experimental steps above were conducted in accordance with the instructions provided in the reagent kit manual.

### 2.9 Determination of AsAb antibody and NO levels

Serum samples were collected from mice on days 0, 7, 14, 21, 35, and 42 after administration, and the levels of AsAb and NO were detected using different ELISA kits according to the manufacturer’s instructions. All the experimental steps above were conducted in accordance with the instructions provided in the reagent kit manual.

In the ELISA experiment, the blood volume of each mouse was (1,100 ± 200) μL, and the serum volume after standing and separation was (450 ± 25) μL, which was detected using a microplate reader. The required mouse materials, dosage, and absorbance for detecting each indicator in 2.7, 2.8, and 2.9 are shown in Table 1.

**Table 1:** The required mouse materials, dosage, and absorbance for detecting each indicator.

Type	Materials	Dosage	Absorbance
MDA	Mouse testicular tissue	100 mg	532 nm
ACP	Mouse testicular tissue	100 mg	520 nm
IgE	Mouse serum	50 μl	450 nm
IgG1	Mouse serum	50 μl	450 nm
IgG2a	Mouse serum	50 μl	450 nm
IFN-γ	Mouse serum	50 μl	450 nm
IL-4	Mouse serum	50 μl	450 nm
IL-6	Mouse serum	50 μl	450 nm
TNF-α	Mouse serum	50 μl	450 nm
NO	Mouse serum	50 μl	450 nm
AsAb	Mouse serum	10 μl	450 nm

### 2.10 Expression analysis of apoptosis-related genes and spermatogenesis-related genes in testicular spermatogenic cells

Total RNA was isolated from the testes of mice in each group by using the RNA extraction kit in accordance with the manufacturer’s instructions. According to the instructions of the kit, the amount of RNA used is 50 ng-2 μg, the total reaction volume is 20 μL, and cDNA reverse transcription is performed under the following conditions: 42 °C for 15 min and 95 °C for 3 min. The obtained cDNA was stored at −20 °C for subsequent analysis. The following mouse gene sequences from GenBank were used for the analysis: Bax, Caspase-3, Bcl-2, p53, c-kit, PlZF, SYCP3, Stra8, Dnajb13, Mrto4, Ipo11, and *β*-action, and the primer sequences and Genbank accession numbers for all genes are listed in Table 2. The corresponding specific primers were designed using Oligo primer analysis software version 7 and synthesized by Kumei Biological Co., Ltd. Quantitative PCR (qPCR) was performed under the following reaction conditions: 95 °C for 10 min; 40 cycles of 95 °C for 10 s, 60 °C for 30 s, and 72 °C for 32 s; and final extension at 55 °C for 40 s, with a temperature change rate of 1 °C/s; the total reaction volume is 50 μL. The fluorescence quantitative PCR instrument is sourced from Analytikjena.

**Table 2:** The primer sequences and Genbank accession numbers for all genes.

Genes	Primer sequences (5′ → 3′)	Genbank accession no.
Bax	F: TGGTTGCCCTCTTCTACTTTGC R: TGTCCAGCCCATGATGGTTC	NM_001411996.1
Caspase-3	F: TGGAATGTCATCTCGCTCT R: ACCATGGCTTAGAATCACAC	NM_001284409.1
Bcl-2	F: CTACCGTCGTGACTTCGCAGA R: ACACATGACCCCACCGAAC	NM_177410.3
p53	F: TGAACCGCCGACCTATCCTTACC R: CTAGGCTGGAGGCTGGAGTGAG	NM_001127233.2
c-kit	F: CACTCACGGGCGGATCACAAA R: CCACTTCACGGGCAGTCGTGC	NM_021099.3
PlZF	F: TCAATGCGGTGCCCAGTTCTCA R: AGTGCGCTTTGTGCCTGAAAGC	NM_001410497.1
SYCP3	F: GGGGCCGGACTGTATTTACT R: AGGCTGATCAACCAAAGGTG	NM_011517.2
Stra8	F: ACAACCTAAGGAAGGCAGTTTAC R: GACCTCCTCTAAGCTGTTGGG	NM_009292.3
Dnajb13	F: AACTTGCCCTGAAGAACCACC R: CCTTCCTCACCAAACTTGTCAT	XM_036153418.1
Mrto4	F: GATAGAAGAGCTTCGGAAATGTGTG R: TTTGCCAAAGAACATCCGGC	NM_001290810.1
Ipo11	F: AGCACGTTTCTTCTGGCAAT R: CTTCCGCAGCACTTTTAACGA	NM_029665.4
β-actin	F: CATCCGTAAAGACCTCTATGCCAAC R: ATGGAGCCACCGATCCACA	NM_007393.5

### 2.11 Statistical analysis

The experimental data were analyzed using SPSS 19.0 and expressed as mean ± standard deviation (^−^X ± SD). Shapiro Wilk test and Levene test were used, respectively, to check the normality and homogeneity of variance of the data. Meet the normal distribution. Analyze the data differences within and between groups using one-way ANOVA and two-way ANOVA. *p* < 0.05 is statistically significant.

## 3 Results

### 3.1 Sperm morphology determined by the modified PAP staining method

**Figure 2:**
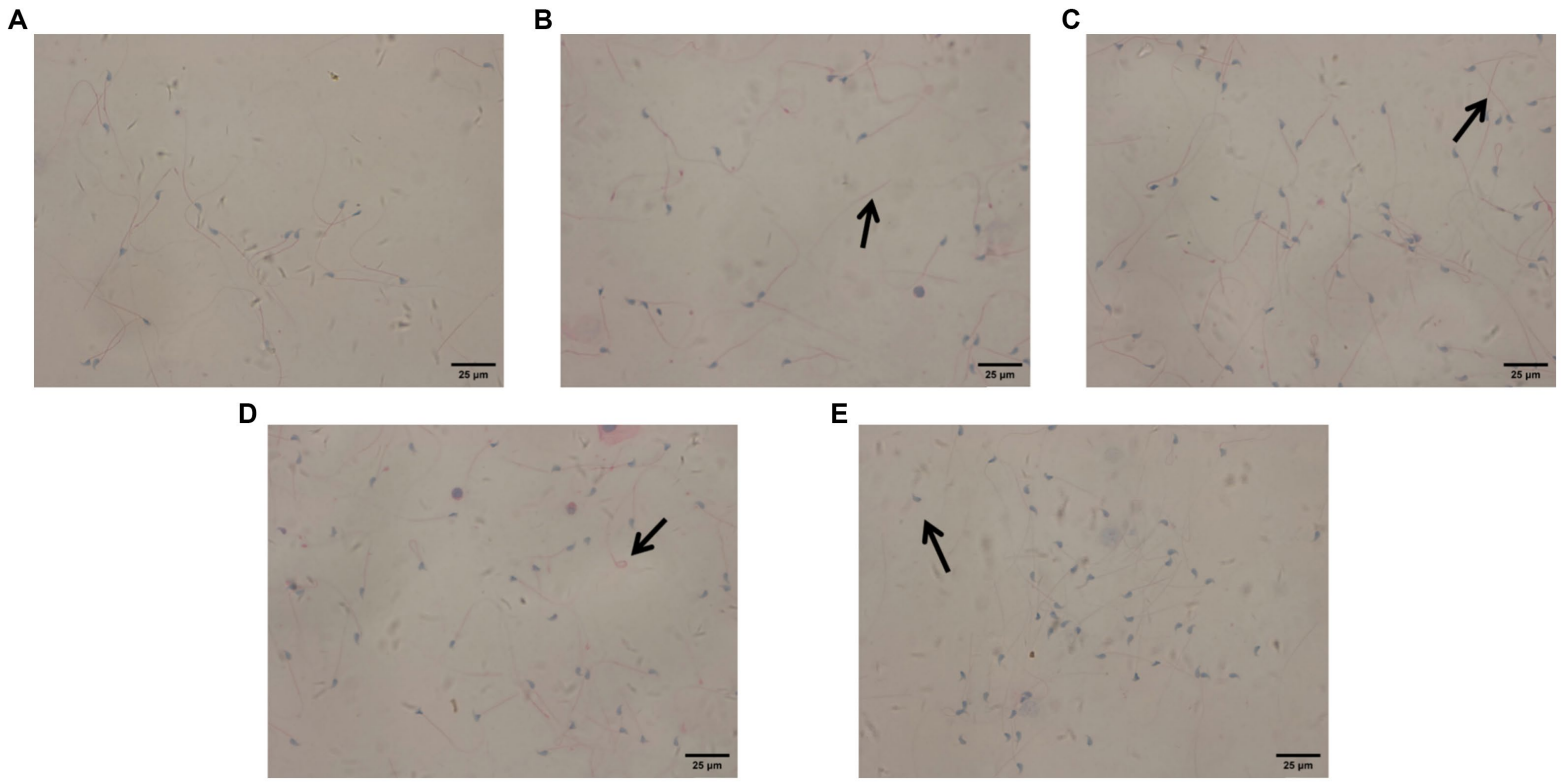
Observation of sperm morphology in male mice (×100). The arrows indicate all abnormal sperm. **(A)** Blank control group. **(B)** Model group. **(C)** *Inonotus obliquus* polysaccharide group. **(D)** Artemisinin group. **(E)** Dihydroartemisinin group.

**Figure 3:**
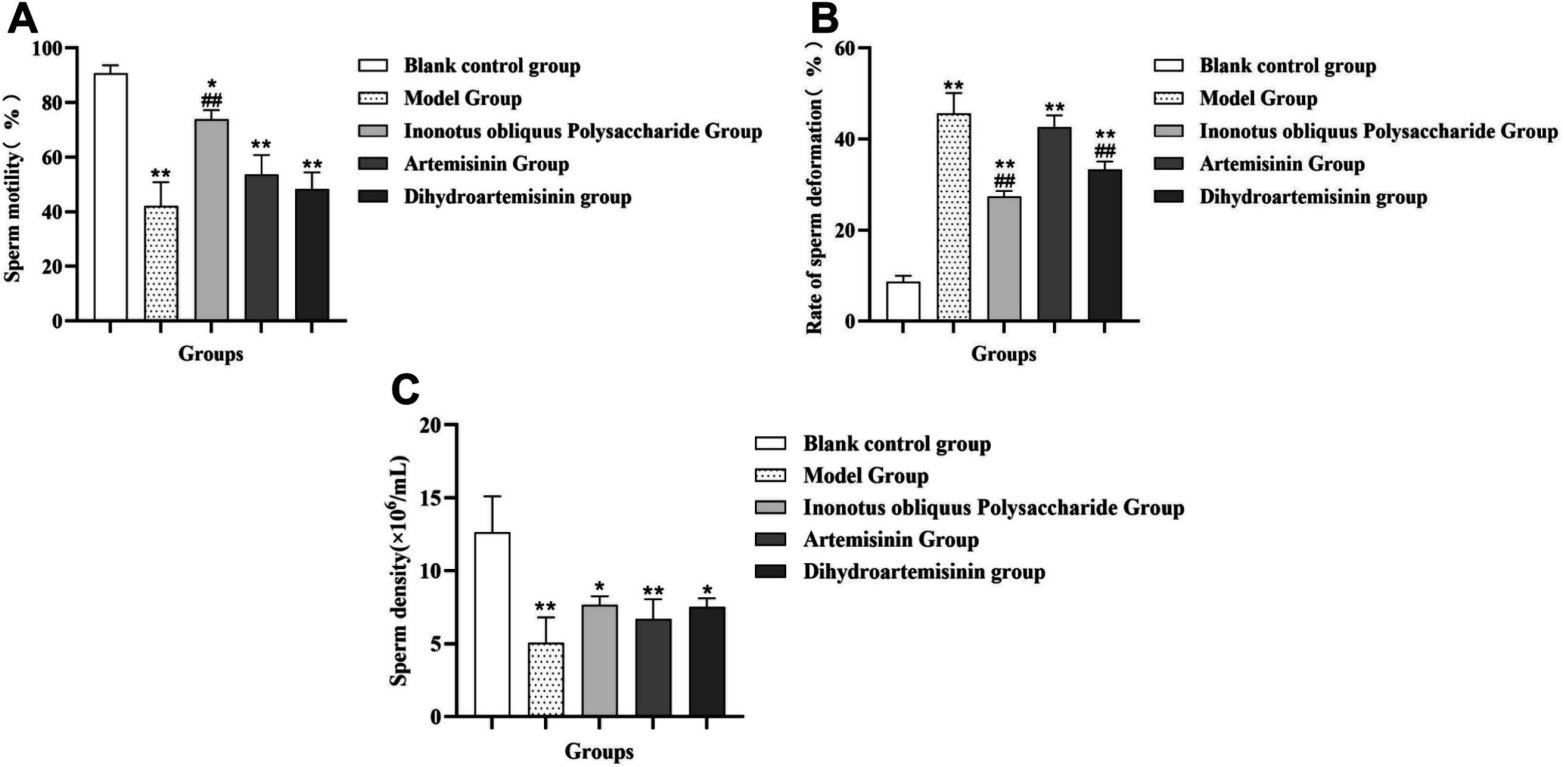
Sperm quality analysis. Each group of mice consists of 5 mice, and the tissue used is the unilateral epididymis from each mouse. After being prepared into a sperm suspension, it can be used to measure the above three sets of data and perform PAP. **(A)** Results of sperm motility in male mice. **(B)** Results of sperm deformity rate in male mice. **(C)** Results of sperm density in male mice. **Compared with the blank control group, *p* < 0.01; *Compared with the blank control group, *p* < 0.05. ^##^Compared with the model group, *p* < 0.01. ^#^Compared with the model group, *p* < 0.05.

The blank control group showed only a small number of abnormal sperm (Figure 2A); in contrast, the infected model group had a large number of abnormal sperm (Figure 2B). Among the three treatment groups, the ART-treated group had the largest number of abnormal sperm (Figure 2D), followed by the DHA-treated group (Figure 2E), while the IOPs-treated group had the smallest number of abnormal sperm (Figure 2C). The sperm deformity rate of each group is shown in Figure 3B.

The sperm motility of the blank control group, model group, Polyporus obliquus polysaccharide group, artemisinin group, and dihydroartemisinin group was (90.67 ± 3.050)%, (42.09 ± 8.760)%, (73.95 ± 3.330)%, (53.60 ± 7.190)%, and (48.35 ± 6.100)%, respectively. The sperm malformation rates of the blank control group, model group, Polyporus obliquus polysaccharide group, artemisinin group, and dihydroartemisinin group were (8.650 ± 1.330)%, (45.65 ± 4.500)%, (27.40 ± 1.223)%, (42.67 ± 2.560)%, and (33.33 ± 1.750)%, respectively. The sperm densities of the blank control group, model group, Polyporus obliquus polysaccharide group, artemisinin group, and dihydroartemisinin group were (12.65 ± 2.450) × 10^6^/mL, (5.05 ± 1.750) × 10^6^/mL, (7.65 ± 0.600) × 10^6^/mL, (6.70 ± 1.350) × 10^6^/mL, and (7.52 ± 0.5965) × 10^6^/mL, respectively.

### 3.2 Sperm quality analysis

#### 3.2.1 Sperm motility

Compared with the blank control group, the sperm motility of mice in the model group, IOPs-treated groups (*p* < 0.0001), ART-treated groups (*p* = 0.0451), and DHA-treated groups (*p* = 0.0002) was significantly reduced. Compared with the model group, the sperm motility of mice in the IOPs-treated groups (*p* = 0.0006) was significantly increased (Figure 3A).

#### 3.2.2 Sperm deformity rate

Compared with the blank control group, the sperm malformation rate in the model group and the drug administration group was significantly increased (*p* < 0.0001). Compared with the model group, the sperm malformation rate in the IOPs-treated groups (*p* < 0.0001) and DHA-treated groups (*p* = 0.0012) was significantly decreased (Figure 3B).

#### 3.2.3 Sperm density

Compared to the blank control group, the sperm count of mice in the model group (*p* = 0.0008), IOPs-treated groups (*p* = 0.0102), ART-treated groups (*p* = 0.0052), and DHA-treated groups (*p* = 0.0138) was significantly reduced. However, there was no significant difference in sperm count between the drug-treated group and the model group (*p* > 0.05) (Figure 3C).

### 3.3 Reproductive organ index

The following results were obtained after statistical analysis.

**Figure 4:**
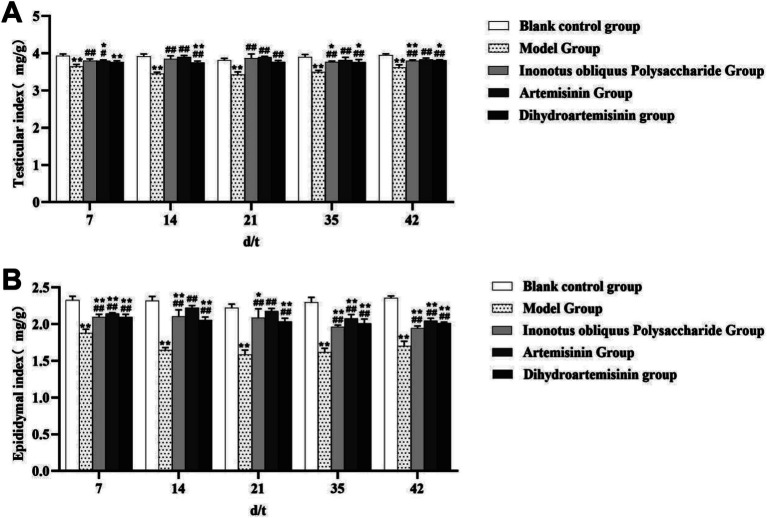
Changes in reproductive organ indices of male mice in each experimental group. **(A)** Testicular index. **(B)** Epididymis index. ^**^Compared with the blank control group, *p* < 0.01; *ompared with the blank control group, *p* < 0.05. ##Compared with the model group, *p* < 0.01. #Compared with the model group, *p* < 0.05.

Testicular index: The blank control and infected model groups showed significant decrease in the testicular index on the 7th, 14th, 21st, 35th, and 42nd days after treatment (*p* < 0.01). Specifically, the infected model and IOPs-treated groups showed significant increase in the testicular index on the 7th, 14th, 21st, 35th, and 42nd days after treatment (*p* < 0.01). The infected model and ART-treated groups showed a significant increase in the testicular index on the 7th day after treatment (*p* < 0.05), which further increased on the 14th, 21st, 35th, and 42nd days after treatment (*p* < 0.01). The infected model and DHA groups showed no significant difference in the testicular index on the 7th day after treatment (*p* > 0.05), but which increased significantly on the 14th, 21st, 35th, and 42nd days after treatment (*p* < 0.01; Figure 4A).

In the blank control group, the testicular index of mice showed no significant trend from day 7 to day 42. In the model group, the testicular index exhibited a pattern of initial decrease followed by an increase, with notable fluctuations during the first 21 days—declining from (3.649 ± 0.054) mg/g to (3.432 ± 0.049) mg/g, and then rising to (3.622 ± 0.028) mg/g by day 42. In the *Inonotus obliquus* polysaccharide group, the testicular index demonstrated an initial increase followed by a decrease, also with clear fluctuations in the first 21 days—rising from (3.803 ± 0.043) mg/g to (3.868 ± 0.114) mg/g, then dropping to (3.793 ± 0.030) mg/g by day 42. In the artemisinin group, a similar initial increase followed by a decrease was observed, with marked fluctuations within the first 14 days—increasing from (3.797 ± 0.025) mg/g to (3.908 ± 0.034) mg/g, before declining to (3.834 ± 0.039) mg/g at day 42. In contrast, the dihydroartemisinin group displayed a slight but steady increasing trend, with the testicular index gradually rising from (3.760 ± 0.037) mg/g on day 7 to (3.809 ± 0.016) mg/g by day 42.

Epididymis index: The blank control and infected model groups showed significant decrease in the epididymis index on the 7th, 14th, 21st, 35th, and 42nd days after treatment (*p* < 0.01). Moreover, all three treatment groups when comparison with infected group showed highly significant increase in on the 7th, 14th, 21st, 35th, and 42nd days after treatment (*p* < 0.01; Figure 4B).

In the blank control group, the epididymal index of mice showed no significant trend from day 7 to day 42. The model group exhibited an initial decrease followed by an increase, with notable fluctuations during the first 21 days—declining from (1.883 ± 0.047) mg/g to (1.583 ± 0.064) mg/g, before rising to (1.703 ± 0.064) mg/g by day 42. The *Inonotus obliquus* polysaccharide group demonstrated an increase followed by a decrease. Marked fluctuations were observed in the first 14 days, with values rising from (2.098 ± 0.037) mg/g to (2.197 ± 0.089) mg/g. From day 21 onward, the index declined, reaching (1.946 ± 0.031) mg/g by day 42. The artemisinin group showed a similar pattern of an initial rise and subsequent fall. Prominent fluctuations occurred within the first 14 days, increasing from (2.145 ± 0.010) mg/g to (2.221 ± 0.032) mg/g. Starting from day 21, the values decreased, falling to (2.049 ± 0.030) mg/g at the end of the treatment period (day 42). In contrast, the dihydroartemisinin group displayed no significant trend in the epididymal index from day 7 to day 42.

### 3.4 Pathological observation

#### 3.4.1 Histopathological changes in the testes

**Figure 5:**
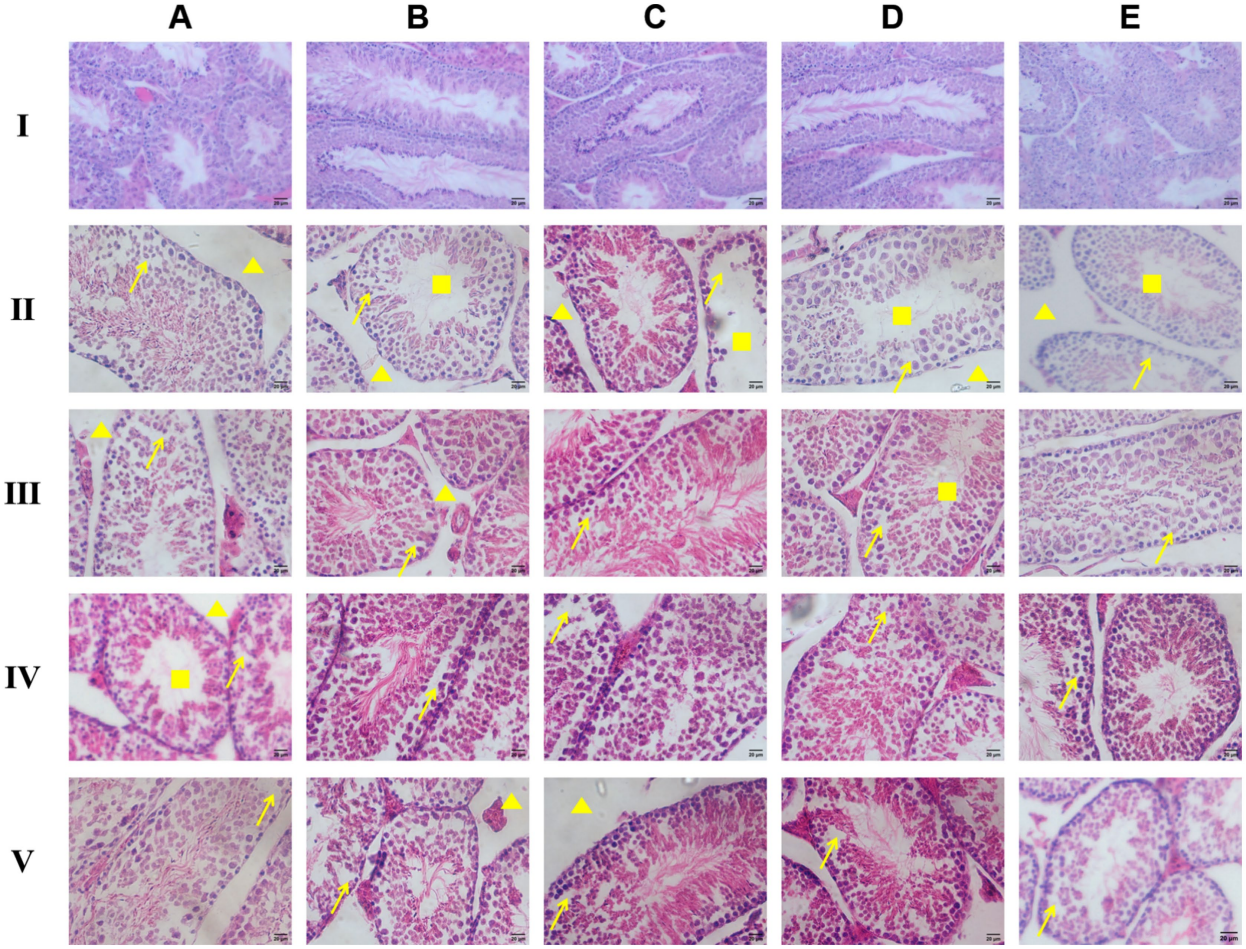
Observation of HE staining of pathological sections of male rat testicular tissue (×200). → refers to the disorderly arrangement of spermatogenic cells; ▲ refers to the decrease in the number of stromal cells between seminiferous tubules; ■ refers to the decrease in sperm count **I** the blank control group, **II** the model group, **III** the IOPs-treated groups, **IV** the ART-treated groups, **V** the DHA-treated groups; **(A)** the 7th day of drug administration, **(B)** the 14th day, **(C)** the 21st day, **(D)** the 35th day, **(E)** the 42nd day.

Compared to the blank control group, the infected model group exhibited pathological changes in the testicular tissue on each test day. The arrangement of spermatogenic cells in all layers of the seminiferous epithelium became disordered, the number of interstitial cells between the seminiferous tubules decreased, and the number of sperm in the lumen of the seminiferous tubules declined. On the 7th test day, apparent sperm cell sloughing into lumen was noted, with narrowing of the space between the seminiferous tubules (Figure 5II-A).

Compared to the blank control group, the IOPs-treated group exhibited sperm cell shedding from the seminiferous tubules on the 7th test day (Figure 5III-A). Although the number of sperm in the IOPs-treated group decreased, it was slightly more than that in the infected model group; moreover, there were disorganization of germinal epithelium. From the 14th to 21st test days, the present of sperm in the seminiferous tubules gradually increased (Figures 5III-B/C); however, from the 35th test day, the number of sperm decreased, with shedding of spermatogenic cells and narrowing of the space between the seminiferous tubules (Figure 5III-D).

Compared to the blank control group, the ART-treated group exhibited a reduction in the number of interstitial cells between the seminiferous tubules; moreover, spermatogenic cells in all layers of the seminiferous epithelium were irregularly arranged. On the 7th test day, the number of sperm in the lumen of the seminiferous tubules decreased (Figure 5IV-A), with a slight increase in the number of sperm on the 14th test day (Figure 5IV-B). However, from the 21st test day, spermatogenic cell shedding from the seminiferous tubules was noted, with narrowing of the lumen of the seminiferous tubules (Figure 5IV-C).

Compared to the blank control group, the DHA-treated group showed initially a decrease in the number of sperm, although it was more than that in the infected model group. On the 14th test day, vacuoles appeared in the cytoplasm of spermatogonia at the base of the seminiferous tubules (Figure 5V-B). From the 21st test day, the number of sperm in seminiferous tubules increased, with a decrease in the number of exfoliated spermatogenic cells (Figure 5V-C).

#### 3.4.2 Histopathological changes in the epididymis

**Figure 6:**
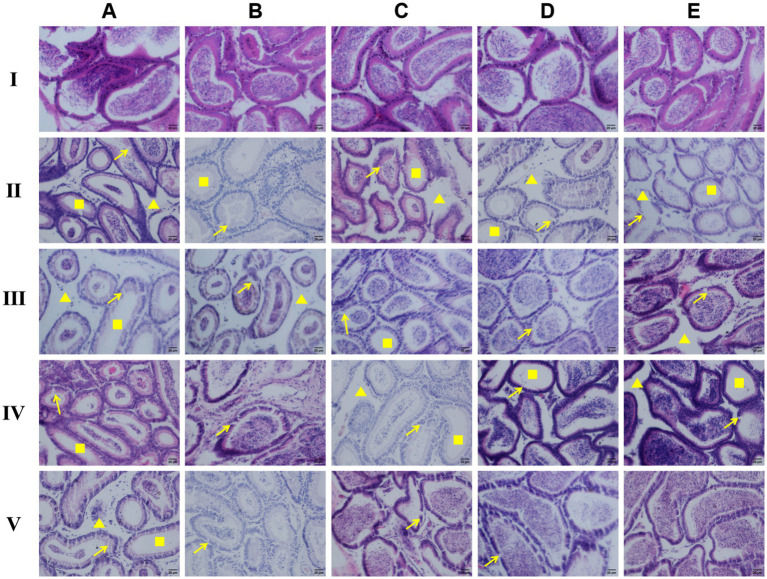
Observation of HE staining of epididymal tissue pathological sections of male rats (×200). → indicates damage to the epithelial cells of the epididymal duct; ▲ indicates a reduction in the number of smooth muscle cells surrounding the epididymal duct; ■ indicates a decrease in the number of mature sperm within the epididymal duct. **I** the blank control group, **II** the model group, **III** the IOPs-treated groups, **IV** the ART-treated groups, **V** the DHA-treated groups; **(A)** the 7th day of drug administration, **(B)** the 14th day, **(C)** the 21st day, **(D)** the 35th day, **(E)** the 42nd day.

From the 7th to the 42nd day after drug administration, compared with the blank control group, the number of mature sperm in the epididymal ducts of the infection model group gradually decreased, the number of epithelial cells in the epididymal ducts reduced, and the gap between the ducts increased. On the 7th test day, the epididymal epithelial cells became atrophic (Figure 6II-A). On the 21st test day, the epididymal tube wall was partially damaged (Figure 6II-B).

Compared to the blank control group, the IOPs-treated group showed a significant reduction in the number of sperm on the 7th test day; however, the number of sperm increased as the test progressed further (Figure 6III-A). The number of smooth muscle cells surrounding the epididymal ducts decreases, and the gap between the ducts widens; moreover, the epithelial cells of the epididymal canals became atrophic between the 21st and 35th test days (Figures 6III-C/D).

Compared to the blank control group, the ART-treated group initially showed a significant decrease in the number of sperm on the 7th test day (Figure 6IV-A), with a subsequent increase in the number of sperm as the test progressed further. From the 21st test day, the number of interstitial cells between the epididymal tubes decreased (Figure 6IV-C). The epididymal canal lumen showed a malformation on the 35th test day (Figure 6IV-D).

Compared to the blank control group, the number of sperm in the DHA-treated group was significantly reduced on the 7th test day (Figure 6V-A), but increased later as the test progressed further. The number of interstitial cells was also substantially decreased on the 7th test day (Figure 6V-A), with an increase in the gap between the epididymal tubes. However, from the 14th test day, the gap gradually became normal (Figure 6V-B).

### 3.5 Transmission electron microscopy (TEM) observation of tissues and organs

#### 3.5.1 TEM observation of brain tissue

**Figure 7:**
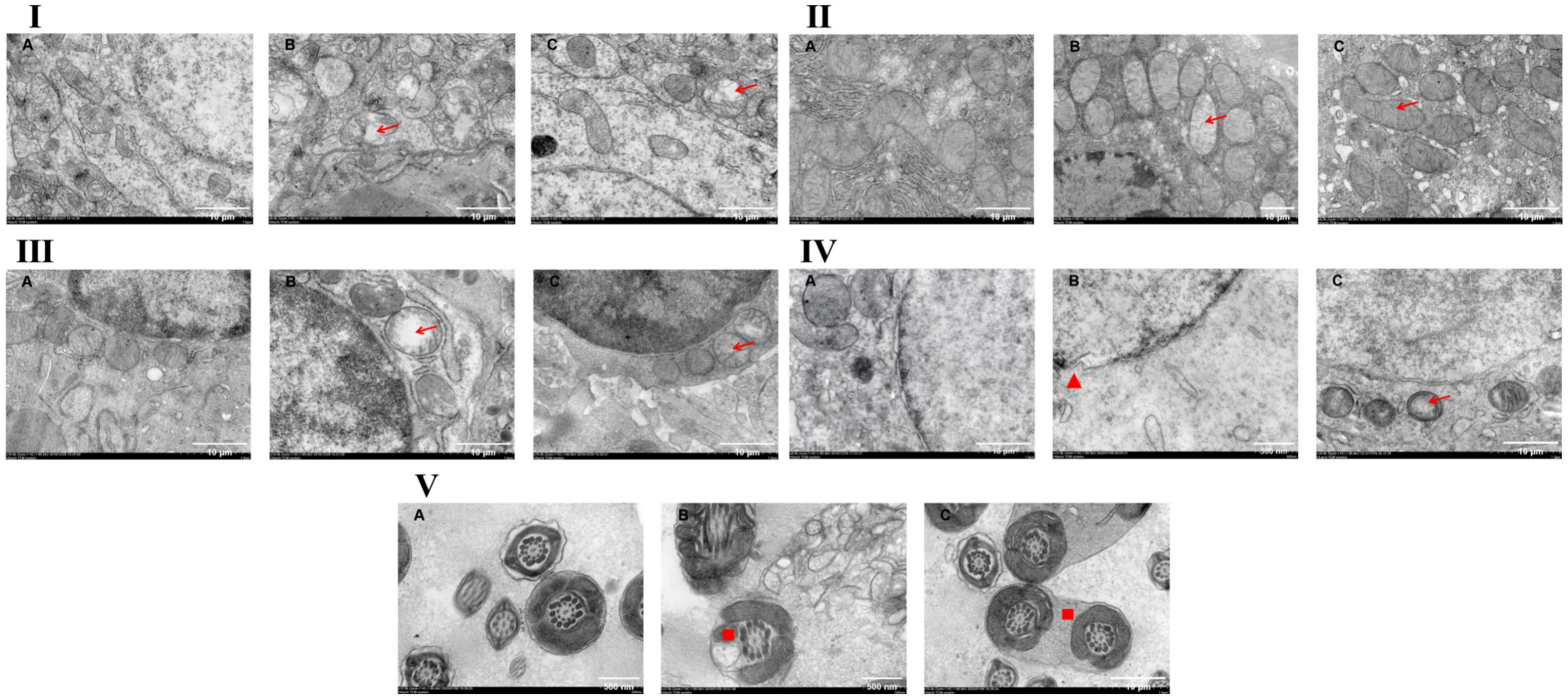
Observation of ultrastructure of various tissues in male mice. → indicates mitochondrial damage; ▲ indicates nuclear membrane damage; ■ indicates malformed sperm. **I** Brain; **II** Liver; **III** Spleen; **IV** Testic; **V** Epididymis. **(A)** Blank control group; **(B)** Model group; **(C)** *Inonotus obliquus* polysaccharide group.

Under electron microscopic examination, the blank control group exhibited a large number of mitochondria with normal morphology, and the nuclear envelope as well as the rough endoplasmic reticulum also appeared normal in structure (Figure 7I-A). In contrast, the model group showed extensive loss of mitochondrial cristae and vacuolization of mitochondria (Figure 7I-B). However, in the *Inonotus obliquus* polysaccharide-treated group, the mitochondria returned to normal with reappearance of cristae, disappearance of vacuolization, and restoration of normal nuclear envelope integrity (Figure 7I-C).

#### 3.5.2 TEM observation of liver tissue

Under electron microscopic examination, the blank control group exhibited abundant rough endoplasmic reticulum and mitochondria, both displaying normal morphology (Figure 7II-A). In contrast, the model group presented with loss of mitochondrial cristae, vacuolization in a subset of mitochondria, a reduced quantity of rough endoplasmic reticulum, invagination of the nuclear membrane, and a decrease in the fibrillar components within the nucleolus (Figure 7II-B). Treatment with *Inonotus obliquus* polysaccharides resulted in the restoration of normal morphology in a large number of mitochondria, although a few vacuoles were observed in the cytoplasm (Figure 7II-C).

#### 3.5.3 TEM observation of spleen tissue

Under electron microscopic examination, the blank control group showed abundant mitochondria, with normal morphology observed in the mitochondria, Golgi apparatus, and nuclear membrane (Figure 7III-A). In the model group, mitochondrial cristae were lost, vacuolization appeared in the mitochondria, and the Golgi apparatus exhibited abnormal morphology, while no significant alterations were noted in the nuclear membrane (Figure 7III-B). In contrast, the *Inonotus obliquus* polysaccharide treatment group displayed partial loss of mitochondrial cristae and vacuolization in some mitochondria, although the nuclear membrane remained normal (Figure 7III-C).

#### 3.5.4 TEM observation of testicular tissue

Under electron microscopic examination, the blank control group exhibited normal structures of mitochondria, nuclear membrane, and rough endoplasmic reticulum, along with normally appearing autophagosomes (Figure 7IV-A). In the model group, the nuclear membrane appeared shrunken and ruptured, mitochondria showed structural abnormalities including loss of cristae and vacuolization, and the cytoplasm displayed a reduced number of organelles (Figure 7IV-B). In the IOP-treated group, the nuclear membrane was shrunken with indistinct boundaries, partial loss of mitochondrial cristae was observed in some mitochondria, and no extensive vacuolization was present (Figure 7IV-C).

#### 3.5.5 TEM observation of epididymis sperm

Under electron microscopic examination, the blank control group displayed normal midpieces and principal pieces of sperm tails, with intact “9 + 2” microtubule doublet arrangement. Occasional wrinkling of the plasma membrane surrounding the principal piece was also observed (Figure 7V-A). In the model group, vacuolization was present in the mitochondria of the sperm midpiece, accompanied by uneven distribution of the outer dense fibers. Oblique sections of sperm tails revealed loss of mitochondrial cristae. Additionally, degenerating cytoplasmic remnants suggestive of autophagic activity (Figure 7V-B). In the IOP-treated group, mild mitochondrial vacuolization was observed; however, malformed spermatozoa exhibiting a biflagellate (two-tailed) structure were also present (Figure 7V-C).

### 3.6 FCM-based detection of spermatogenic cell apoptosis in male mice

**Figure 8:**
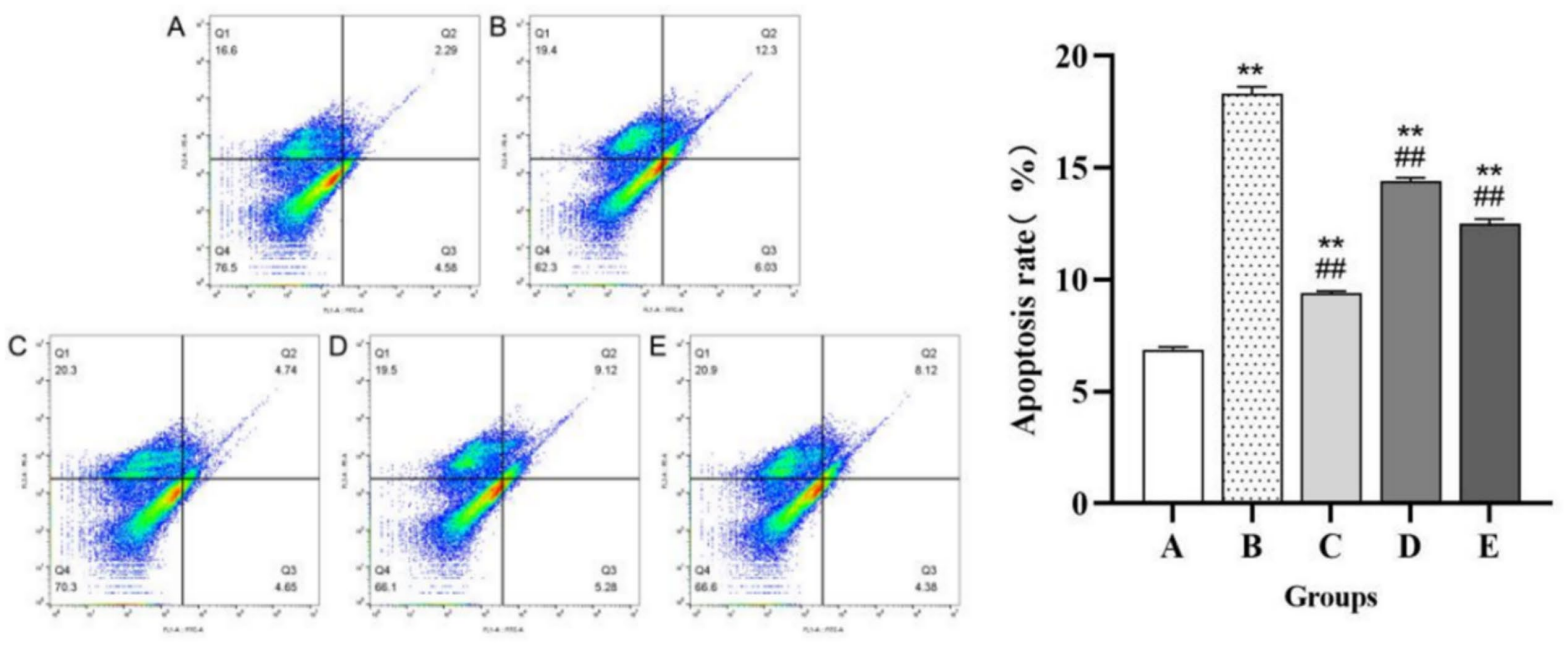
Shows the apoptosis results of spermatogenic cells in male mice of each experimental group. The left figure is a flow cytometry diagram of cell apoptosis, where Q2 and Q3 represent late-stage apoptotic cells and early-stage apoptotic cells, respectively. The right figure shows the percentage of cell apoptosis, which is the percentage of the total number of early-stage and late-stage apoptotic cells out of all cells. **(A)** Blank control group. **(B)** Model group. **(C)** *Inonotus obliquus* polysaccharide group. **(D)** Artemisinin group. **(E)** Dihydroartemisinin group. Compared with the blank control group, *p* < 0.01; *Compared with the blank control group, *p* < 0.05. ##Compared with the model group, *p* < 0.01. #Compared with the model group, *p* < 0.05.

As shown in Figure 8, the blank control and infected model groups exhibited a highly significant difference in the level of spermatogenic cell apoptosis (*p* < 0.01). Similarly, all three treatment groups and the infected model group showed a highly significant difference in the level of spermatogenic cell apoptosis (*p* < 0.01).

The spermatogenic cell apoptosis rates showed significant differences among the experimental groups. As expected, the model group exhibited a markedly increased apoptosis rate of (18.330 ± 0.300)%, compared to (6.867 ± 0.125)% in the blank control group. Among the treatment groups, the *Inonotus obliquus* polysaccharide group demonstrated the lowest rate at (9.390 ± 0.100)%, followed by the dihydroartemisinin group at (12.500 ± 0.200)% and the artemisinin group at (14.400 ± 0.150)%, all of which were lower than that of the model group.

### 3.7 Determination of MDA levels in the testicular tissue of male mice by the thiobarbituric acid method

**Figure 9:**
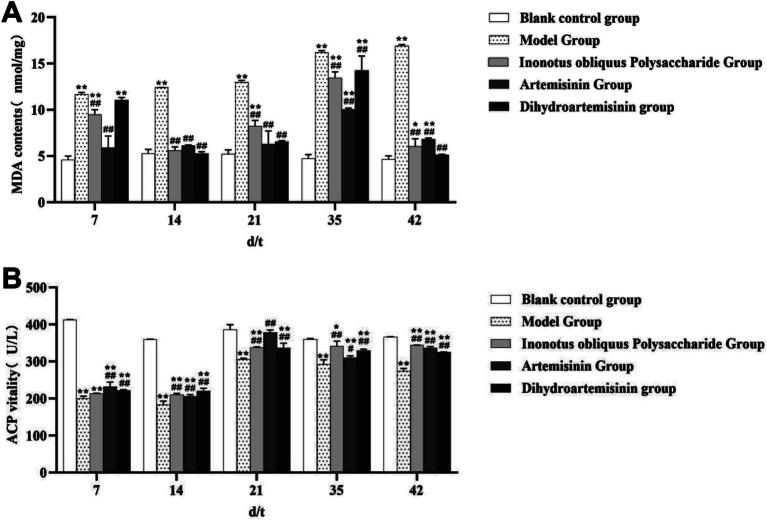
Oxidation level analysis. **(A)** Changes in MDA content in mouse testicular tissue. **(B)** Changes in ACP content in mouse testicular tissue. Compared with the blank control group, *p* < 0.01; *Compared with the blank control group, *p* < 0.05. ##Compared with the model group, *p* < 0.01. #Compared with the model group, *p* < 0.05.

The blank control and model groups showed a highly significant increase (*p* < 0.01) in MDA levels on the 7th, 14th, 21st, 35th, and 42nd test days. The MDA level of the infected model group and those of the IOPs-treated and ART-treated groups showed significant reduction on the 7th, 14th, 21st, 35th, and 42nd test days (*p* < 0.01). Although the MDA levels of the DHA-treated and infected model groups showed no significant difference on the 7th test day (*p* > 0.05), significant reduction were noted on the 14th, 21st, 35th, and 42nd test days (*p* < 0.01; Figure 9A).

The MDA concentrations in testicular tissue exhibited distinct temporal patterns across the experimental groups. In the blank control group, MDA levels showed a minor transient increase followed by a return to baseline, rising from (4.622 ± 0.392) nmol/mg to (5.281 ± 0.448) nmol/mg before declining to (4.633 ± 0.393) nmol/mg. In contrast, the model group displayed a progressive increase in MDA concentration, from (11.670 ± 0.214) nmol/mg on day 7 to (16.910 ± 0.160) nmol/mg by day 42. The *Inonotus obliquus* polysaccharide group demonstrated a more complex trend: MDA levels initially decreased from (11.670 ± 0.214) nmol/mg on day 7 to (5.613 ± 0.368) nmol/mg by day 14, then increased to (13.450 ± 0.666) nmol/mg on day 35, before finally falling to (6.044 ± 0.837) nmol/mg on day 42. The artemisinin group exhibited an initial increase followed by a decrease. After noticeable fluctuations during the first 35 days—rising from (5.992 ± 1.246) nmol/mg to (10.040 ± 0.163) nmol/mg—the concentration declined to (6.820 ± 0.134) nmol/mg by day 42. Similarly, the dihydroartemisinin group also showed a pattern of decrease, increase, and subsequent decrease, with marked early fluctuations. Levels dropped from (11.05 ± 0.281) nmol/mg on day 7 to (5.280 ± 0.197) nmol/mg by day 14, gradually rose to (14.26 ± 1.571) nmol/mg on day 35, and finally decreased to (5.164 ± 0.023) nmol/mg by the end of the treatment period (day 42).

### 3.8 Spectrophotometric determination of ACP activity in the testicular tissue of male mice

ACP activity in testicular tissue showed highly significant reduction between the blank control and infected model groups on the 7th, 14th, 21st, 35th, and 42nd test days (*p* < 0.01). Although the infected model and IOPs-treated groups showed no significant difference in ACP activity on the 7th test day (*p* > 0.05), significant increase were noted on the 14th, 21st, 35th, and 42nd test days (*p* < 0.01). The ACP activity showed a significant increase between the infected model and ART-treated groups on the 7th, 14th, 21st, 35th, and 42nd test days (*p* < 0.05 for the 35th day and *p* < 0.01 for the remainder). Significant increase in ACP activity between the infected model and DHA-treated groups were noted on the 7th, 14th, 21st, 35th, and 42nd test days (*p* < 0.01; Figure 9B).

The concentration of acid phosphatase (ACP) in testicular tissue showed distinct temporal patterns across the experimental groups. In the blank control group, ACP levels initially decreased, then increased, and finally stabilized, with notable fluctuations observed within the first 14 days. Specifically, the concentration declined from (412.91 ± 0.890) U/L to (359.60 ± 1.196) U/L, subsequently rose to (385.8 ± 13.76) U/L, and eventually decreased to (366.0 ± 0.382) U/L before plateauing. In the model group, ACP concentration followed a pattern of initial decrease, subsequent increase, and final decline. From day 7 (200.03 ± 6.412) U/L, it dropped to (183.0 ± 9.652) U/L by day 14, increased to (305.9 ± 3.201) U/L on day 21, and finally decreased to (274.4 ± 6.834) U/L. The *Inonotus obliquus* polysaccharide group exhibited a gradual increase in ACP concentration, rising from (213.64 ± 0.701) U/L to (344.4 ± 0.154) U/L over the experimental period. In the artemisinin group, ACP levels showed an initial rise followed by a decline, with considerable fluctuations during the first 21 days. The concentration increased from (232.00 ± 12.440) U/L to (379.03 ± 6.248) U/L and later decreased to (337.12 ± 3.716) U/L. Similarly, the dihydroartemisinin group displayed an initial increase followed by a decrease, with marked fluctuations in the first 21 days. ACP levels rose from (222.33 ± 1.544) U/L to (336.90 ± 12.97) U/L and subsequently stabilized.

### 3.9 ELISA-based quantification of serum levels of IgGs, cytokines, NO, and AsAb

#### 3.9.1 Detection of serum IgG levels

Serum IgE levels in mice exhibited distinct temporal dynamics across the experimental groups. In the blank control group, no significant trend in IgE levels was observed. In contrast, the model group demonstrated an initial decrease, followed by an increase and a subsequent decline: levels dropped from (17.65 ± 0.454) μg/L to (16.46 ± 0.479) μg/L during the first 14 days, rose to (17.46 ± 0.405) μg/L by day 21, and eventually stabilized at (16.14 ± 1.464) μg/L. The *Inonotus obliquus* polysaccharide group displayed an initial increase followed by a decrease, with marked variations in the first 14 days—rising from (15.53 ± 0.971) μg/L to (17.62 ± 0.259) μg/L—before gradually declining to (16.14 ± 1.464) μg/L and stabilizing. In the artemisinin group, IgE levels showed a slight rise followed by a decline, increasing from (17.29 ± 0.263) μg/L to (17.71 ± 0.127) μg/L and then gradually decreasing to (16.43 ± 0.307) μg/L. Similarly, the dihydroartemisinin group exhibited a pattern of modest increase, decrease, and final rise: levels increased from (15.09 ± 0.486) μg/L to (15.34 ± 0.358) μg/L, then declined to (14.17 ± 0.183) μg/L, and finally rose again to (14.87 ± 0.133) μg/L.

According to ELISA results, IgE, IgG1, and IgG2a levels were higher in the infected model and treatment groups than in the blank control group, and these levels continuously increased with the increase in test duration.

From test days 7 to 42, the serum IgE antibody level was significantly higher in the infected model group than in the blank control group (*p* < 0.01). On the 7th and 21st test days, the serum IgE antibody level was significantly higher in the IOPs-treated group than in the infected model group (*p* < 0.05 or *p* < 0.01, respectively). On the 35th test day, the DHA-treated group showed a significantly higher serum IgE antibody level than the infected model group (*p* < 0.01). No significant differences in serum IgE antibody levels were observed between the infected model and ART-treated groups.

Serum IgG1 levels in mice demonstrated distinct temporal patterns across the treatment groups. No significant trend was observed in the blank control group. In the model group, IgG1 levels showed a gradual increase from (8.82 ± 0.035) mg/mL to (12.67 ± 0.049) mg/mL. The *Inonotus obliquus* polysaccharide group exhibited an initial increase followed by a decrease, with notable fluctuations during the first 35 days: levels rose from (9.02 ± 0.014) mg/mL to (14.57 ± 0.018) mg/mL and then declined to (12.94 ± 0.731) mg/mL by day 42. In the artemisinin group, a steady increasing trend was observed, with IgG1 levels rising from (9.324 ± 0.016) mg/mL to (12.82 ± 0.028) mg/mL. Similarly, the dihydroartemisinin group displayed an initial rise followed by a decline. Pronounced fluctuations were noted in the first 35 days, during which levels increased from (9.06 ± 0.052) mg/mL to (14.53 ± 0.018) mg/mL, before decreasing to (13.76 ± 0.018) mg/mL by the end of the treatment period (day 42).

The IOPs-treated group exhibited a significantly higher serum IgG1 antibody level than the infected model group from test days 21 to 35 (*p* < 0.01). The serum IgG1 antibody level of the ART-treated group was significantly higher than that of the infected model group from test days 14 to 35 (*p* < 0.01). The DHA-treated group showed a significantly higher serum IgG1 antibody level than the infected model group from test days 21 to 42 (*p* < 0.01).

Serum IgG2a levels in mice displayed the following trends across experimental groups. No significant change was observed in the blank control group. In contrast, all treatment groups exhibited a clear upward trend in IgG2a levels: the model group increased from (193.21 ± 0.266) μg/mL to (610.19 ± 1.298) μg/mL; the *Inonotus obliquus* polysaccharide group rose from (213.23 ± 0.202) μg/mL to (673.89 ± 1.019) μg/mL; the artemisinin group increased from (209.64 ± 0.431) μg/mL to (645.57 ± 0.531) μg/mL; and the dihydroartemisinin group showed an increase from (255.20 ± 0.290) μg/mL to (679.31 ± 1.497) μg/mL.

**Figure 10:**
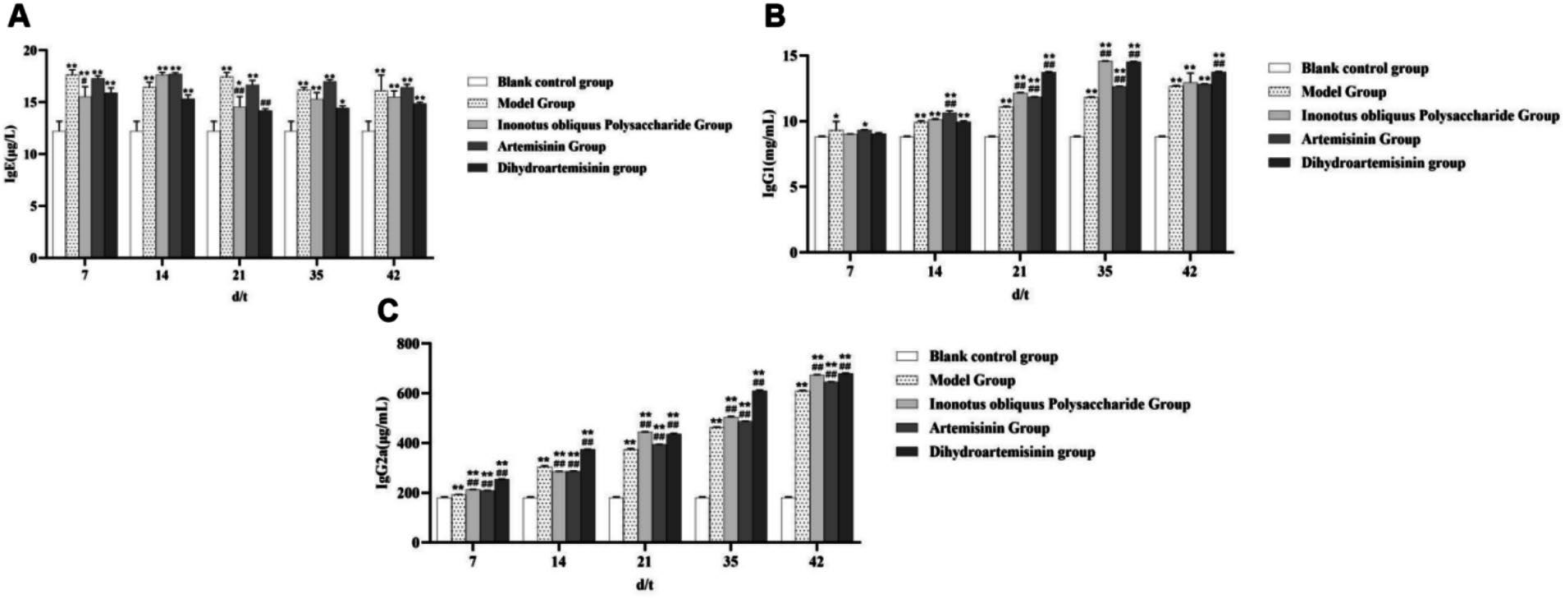
Detection of serum immunoglobulin. **(A)** Changes in IgE concentration in mouse serum. **(B)** Changes in IgG1 concentration in mouse serum. **(C)** Changes in IgG2a concentration in mouse serum. Compared with the blank control group, *p* < 0.01; *Compared with the blank control group, *p* < 0.05. ##Compared with the model group, *p* < 0.01. #Compared with the model group, *p* < 0.05.

From test days 7 to 42, mice in all three treatment groups showed a significantly higher serum IgG2a antibody level than mice in the infected model group (*p* < 0.01; Figure 10).

#### 3.9.2 Detection of serum cytokine levels

In the control group, serum IFN-*γ* levels in mice showed no significant trend of change. In the model group, serum IFN-γ levels exhibited an initial decrease followed by an increase, with notable fluctuations during the first 14 days, declining from (915.5 ± 9.999) ng/L to (850.51 ± 9.707) ng/L, and then gradually rising to (879.72 ± 4.357) ng/L by day 42 of administration. In the *Inonotus obliquus* polysaccharide group, serum IFN-γ levels displayed a similar pattern of initial decrease followed by an increase, with marked fluctuations up to day 35, decreasing from (732.03 ± 27.780) ng/L to (377.28 ± 1.762) ng/L, and then slightly increasing to (405.54 ± 8.866) ng/L by day 42. In the artemisinin group, serum IFN-γ levels also demonstrated an initial decline followed by a rise, with significant variations within the first 14 days, dropping from (711.60 ± 24.89) ng/L to (443.91 ± 10.280) ng/L, and thereafter slowly increasing to (573.47 ± 7.569) ng/L by day 42. In the dihydroartemisinin group, serum IFN-γ levels showed a comparable trend of initial reduction followed by a gradual increase, with pronounced fluctuations during the first 21 days, declining from (609.89 ± 4.465) ng/L to (342.31 ± 1.242) ng/L, and then slowly rising to (437.80 ± 6.081) ng/L by day 42.

No significant trend in serum IL-4 levels was observed in the blank control group. In the model group, serum IL-4 levels showed an initial decrease followed by a subsequent increase, declining from (33.67 ± 0.614) ng/L on day 7 to (21.56 ± 0.120) ng/L on day 21, and then gradually rising to (31.91 ± 0.271) ng/L by day 42 of administration. In the *Inonotus obliquus* polysaccharide group, serum IL-4 levels exhibited a pattern of decrease, followed by an increase, and then a slight decline: from (27.94 ± 0.159) ng/L on day 7 to (14.75 ± 0.030) ng/L on day 14, increasing to (25.28 ± 0.350) ng/L by day 35, and then decreasing slightly to (21.89 ± 0.071) ng/L by day 42. In the artemisinin group, serum IL-4 levels demonstrated an initial reduction followed by a gradual recovery, with notable fluctuations within the first 21 days: decreasing from (34.08 ± 0.743) ng/L to (20.00 ± 0.339) ng/L by day 21, and then rising to (28.81 ± 0.123) ng/L by day 42. In the dihydroartemisinin group, serum IL-4 levels displayed a consistent downward trend, gradually decreasing from (24.23 ± 0.114) ng/L to (15.75 ± 0.079) ng/L over the observation period.

In the blank control group, no significant trend of change was observed in the serum IL-6 levels of mice. In the model group, serum IL-6 levels exhibited an initial increase, followed by a decrease, and then a subsequent rise: from (133.20 ± 1.105) pg/mL on day 7 to (258.41 ± 2.073) pg/mL on day 14, decreasing to (161.76 ± 1.467) pg/mL on day 21, and then gradually increasing again to (230.71 ± 3.838) pg/mL by day 42. In the *Inonotus obliquus* polysaccharide group, serum IL-6 levels showed a similar pattern of increase, decrease, and then rise: rising gradually from (82.34 ± 3.079) pg/mL to (103.60 ± 1.557) pg/mL over the first 21 days, decreasing to (75.74 ± 2.025) pg/mL on day 35, and then increasing again to (98.11 ± 3.025) pg/mL by day 42. In the artemisinin group, serum IL-6 levels demonstrated an initial increase followed by a decrease, with notable fluctuations during the first 35 days: increasing from (97.93 ± 3.792) pg/mL to (160.30 ± 3.403) pg/mL, and then decreasing to (115.79 ± 2.580) pg/mL by day 42. In the dihydroartemisinin group, serum IL-6 levels also displayed an upward trend followed by a decline: increasing from (106.72 ± 2.561) pg/mL to (131.00 ± 2.576) pg/mL within the first 14 days, and then gradually decreasing to (83.32 ± 2.246) pg/mL by day 42.

No significant trend in serum TNF-*α* levels was observed in the blank control group. In the model group, serum TNF-α levels showed a consistent upward trend, gradually increasing from (299.23 ± 4.684) ng/L to (773.11 ± 5.075) ng/L. In the *Inonotus obliquus* polysaccharide group, serum TNF-*α* levels displayed an unstable fluctuation: increasing from (222.01 ± 1.484) ng/L to (320.21 ± 11.900) ng/L within the first 14 days, decreasing to (196.70 ± 12.002) ng/L on day 21, rising again to (210.89 ± 11.531) ng/L on day 35, and finally declining to (154.90 ± 5.765) ng/L by day 42. In the artemisinin group, serum TNF-α levels exhibited an initial increase, followed by a decrease, and then a subsequent rise, with notable fluctuations during the first 14 days: increasing from (177.21 ± 5.948) ng/L to (419.93 ± 10.691) ng/L, then decreasing to (298.71 ± 8.752) ng/L by day 35, and finally rising again to (497.77 ± 18.803) ng/L. In the dihydroartemisinin group, serum TNF-α levels also demonstrated an unstable trend: rising from (286.21 ± 5.061) ng/L to (502.4 ± 15.990) ng/L within the first 14 days, decreasing to (331.23 ± 12.111) ng/L on day 21, increasing to (382.60 ± 2.078) ng/L on day 35, and then declining to (360.59 ± 17.800) ng/L by day 42.

**Figure 11:**
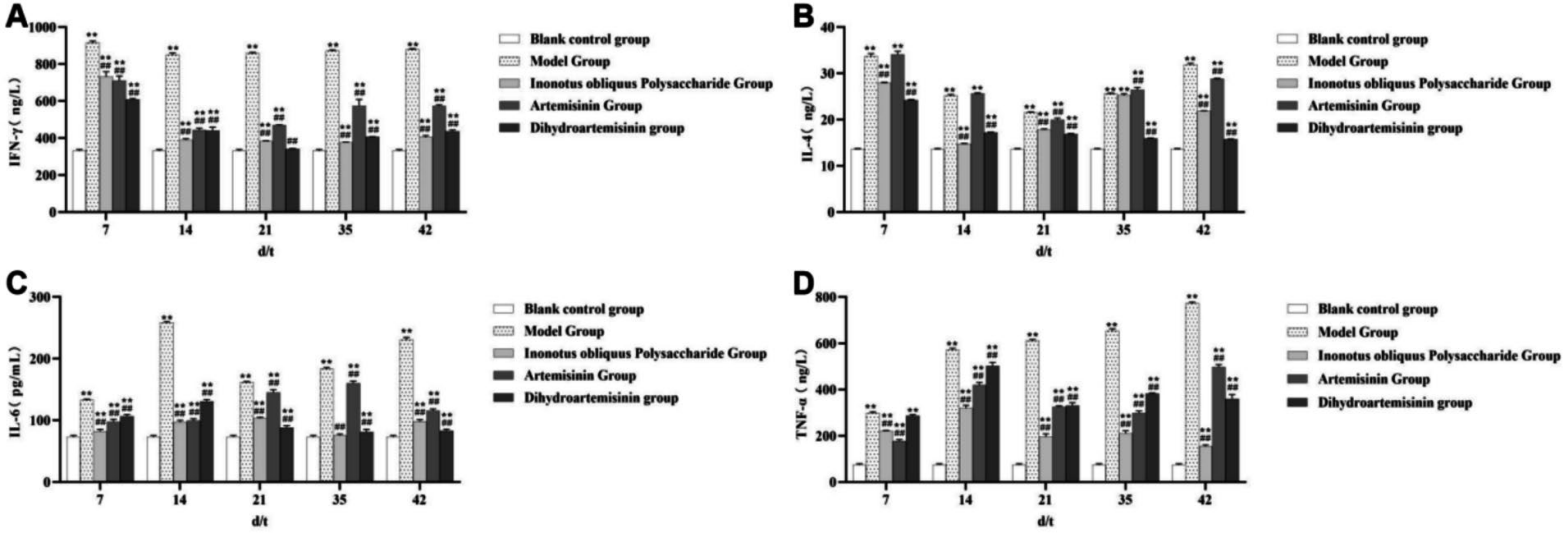
Detection of serum cytokine levels. **(A)** Changes in IFN-*γ* concentration in mouse serum. **(B)** Changes in IL-4 concentration in mouse serum. **(C)** Changes in IL-6 concentration in mouse serum. **(D)** Changes in TNF-α concentration in mouse serum. Compared with the blank control group, *p* < 0.01; *Compared with the blank control group, *p* < 0.05. ##Compared with the model group, *p* < 0.01. #Compared with the model group, *p* < 0.05.

The serum IFN-*γ*, IL-4, IL-6, and TNF-α levels were significantly higher in the infected model group than in the blank control group from test days 7 to 42 (*p* < 0.01). The serum IFN-γ and IL-6 levels of the three treatment groups were significantly lower than that of the infected model group from test days 7 to 42 (*p* < 0.01). Both IOPs-treated and DHA-treated groups exhibited significantly lower serum IL-4 levels than the infected model group from test days 7 to 42 (*p* < 0.01). The serum IL-4 level of the ART-treated group was significantly lower than that of the infected model group from test days 21 to 42 (*p* < 0.01). Both IOPs-treated and ART-treated groups exhibited significantly lower serum TNF-α levels than the infected model group from test days 7 to 42 (*p* < 0.01). The serum TNF-α level was significantly lower in the DHA-treated group than in the infected model group from test days 14 to 42 (*p* < 0.01; Figure 11).

#### 3.9.3 Detection of NO and AsAb levels

No significant trend in serum nitric oxide (NO) concentration was observed in the blank control group. In the model group, serum NO concentration showed a consistent upward trend, gradually increasing from (188.60 ± 0.181) μmol/L to (224.11 ± 0.362) μmol/L. In contrast, serum NO concentration in the *Inonotus obliquus* polysaccharide group exhibited a declining trend, decreasing from (181.12 ± 0.523) μmol/L to (96.64 ± 0.442) μmol/L. Similarly, a decreasing trend was observed in the artemisinin group, with serum NO concentration declining from (186.29 ± 0.895) μmol/L to (118.49 ± 0.428) μmol/L. The dihydroartemisinin group displayed a pattern of initial decrease, followed by a transient increase and a subsequent decline: it decreased from (161.23 ± 0.916) μmol/L to (114.10 ± 0.172) μmol/L within the first 21 days, increased to (139.60 ± 0.220) μmol/L on day 35, and then decreased again to (95.40 ± 0.104) μmol/L by day 42.

No significant trend in serum anti-sperm antibody (AsAb) concentration was observed in the blank control group. In the model group, serum AsAb levels demonstrated an initial increase followed by a subsequent decrease, with notable fluctuations during the first 35 days: increasing from (159.40 ± 0.871) pg/mL to (244.60 ± 3.473) pg/mL, and then declining to (181.70 ± 0.281) pg/mL by day 42. Similarly, in the *Inonotus obliquus* polysaccharide group, serum AsAb concentration showed a pattern of rise followed by decline: increasing from (154.40 ± 2.584) pg/mL on day 7 to (174.90 ± 1.286) pg/mL on day 21, and then decreasing to (148.3 ± 0.793) pg/mL by day 42. In the artemisinin group, serum AsAb levels also exhibited an upward trend initially, followed by a downward change, with marked variations within the first 35 days: rising from (159.00 ± 1.141) pg/mL to (192.70 ± 0.736) pg/mL, and then reducing to (182.10 ± 0.909) pg/mL by day 42. Likewise, the dihydroartemisinin group displayed an initial increase and then a decrease in serum AsAb concentration: from (158.00 ± 1.735) pg/mL on day 7 to (163.40 ± 0.662) pg/mL on day 14, followed by a decline to (143.10 ± 0.228) pg/mL by day 42.

**Figure 12:**
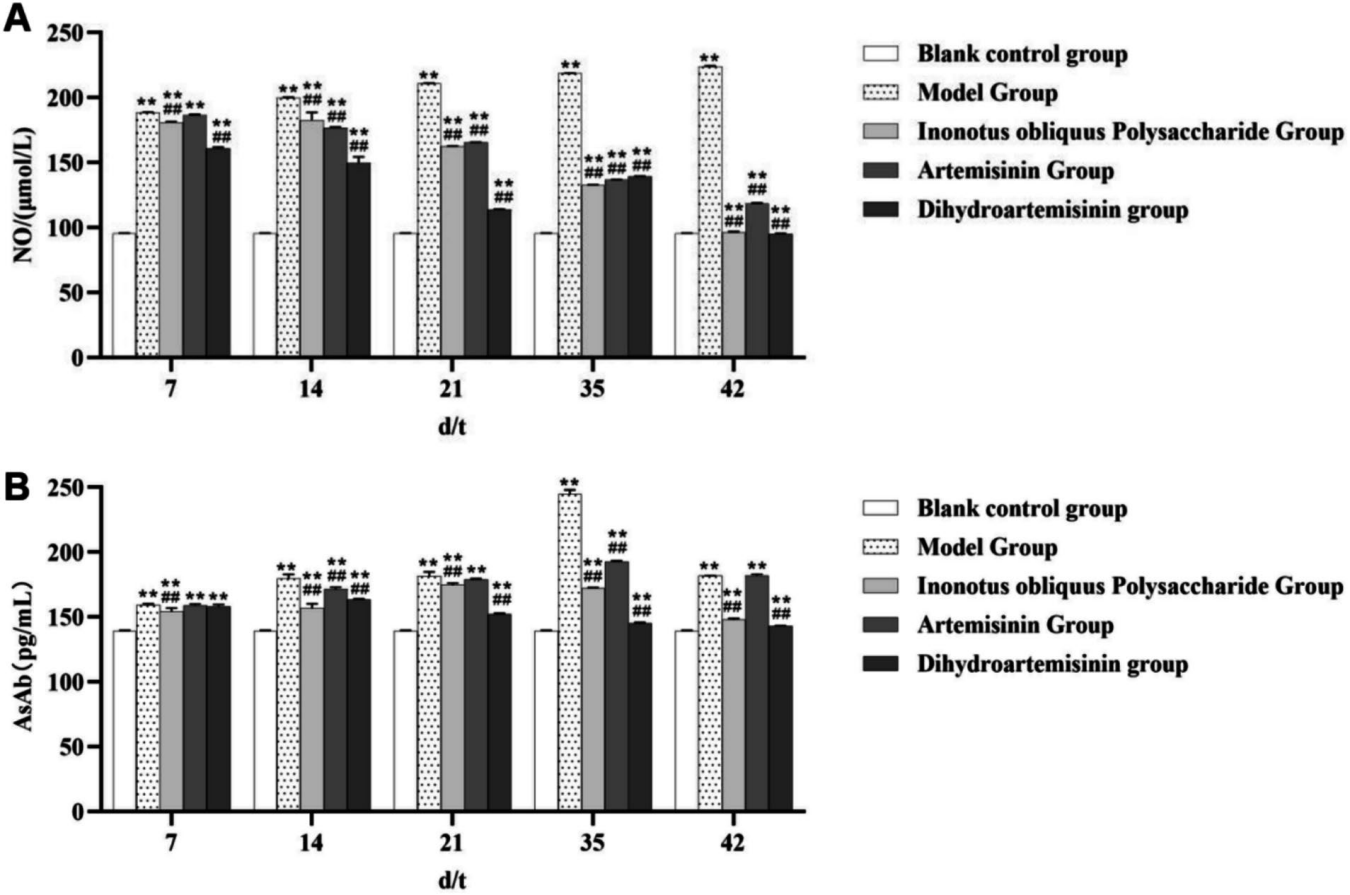
Detection of NO and AsAb levels. **(A)** Changes in NO concentration in mouse serum. **(B)** Changes in AsAb concentration in mouse serum. Compared with the blank control group, *p* < 0.01; ompared with the blank control group, *p* < 0.05. Compared with the model group, *p* < 0.01. Compared with the model group, *p* < 0.05.

The infected model showed significantly higher levels of NO and AsAb than the blank control group (*p* < 0.01). Both the IOPs-treated and DHA-treated groups exhibited significantly lower NO levels than the infected model group from test days 7 to 42 (*p* < 0.01). The NO level of the ART-treated group was significantly lower than that of the infected model group (*p* < 0.01). The AsAb level was significantly lower in the three treatment groups than in the infected model group (*p* < 0.01; Figure 12).

### 3.10 Detection of apoptosis-related genes in testicular spermatogenic cells

#### 3.10.1 Sperm apoptosis gene detection

The mRNA expression level of the apoptosis-related gene Bax was significantly higher in the infected model group than in the blank control group on test days 21 and 42 (*p* < 0.01, respectively). The DHA-treated group showed a significantly lower Bax gene expression than the infected model group from treatment days 21 to 35 (*p* < 0.01).

The Bcl-2 gene expression level was significantly lower in the model group than in the blank control group on the 42nd test day (*p* < 0.01). The DHA-treated group displayed a significantly higher Bcl-2 gene expression level than the infected model group on the 21st test day (*p* < 0.01). The Bcl-2 gene expression level was significantly higher in the ART-treated group than in the infected model group on the 42nd test day (*p* < 0.01).

**Figure 13:**
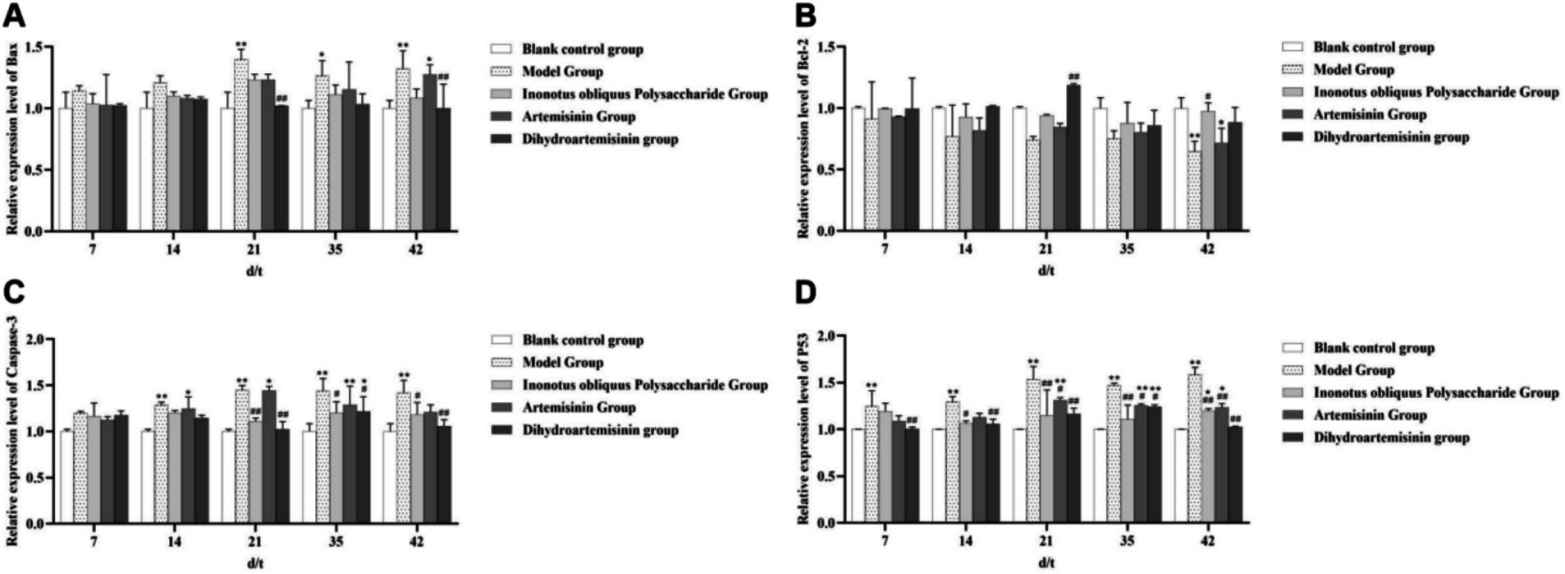
Sperm apoptosis gene detection. **(A)** Expression level of Bax gene in mouse testicular tissue. **(B)** Expression level of Bcl-2 gene in mouse testicular tissue. **(C)** Expression level of Caspase-3 gene in mouse testicular tissue. **(D)** Expression level of P53 gene in mouse testicular tissue. ^**^Compared with the blank control group, *p* < 0.01; *Compared with the blank control group, *p* < 0.05. ##Compared with the model group, *p* < 0.01. #Compared with the model group, *p* < 0.05.

The caspase-3 gene expression level was significantly higher in the infected model group than in the blank control group from test days 14 to 42 (*p* < 0.01). Both IOPs-treated and DHA-treated groups exhibited significantly lower caspase-3 gene expression levels than the infected model group on test days 21 and 42 (*p* < 0.05 respectively). The p53 gene expression level in the model group was significantly higher than that in the blank control group from test days 7 to 42 (*p* < 0.01). All three treatment groups showed significantly lower p53 gene expression levels than the infected model group on test days 7 and 42 (*p* < 0.05; Figure 13).

#### 3.10.2 Genetic testing of spermatogenesis

**Figure 14:**
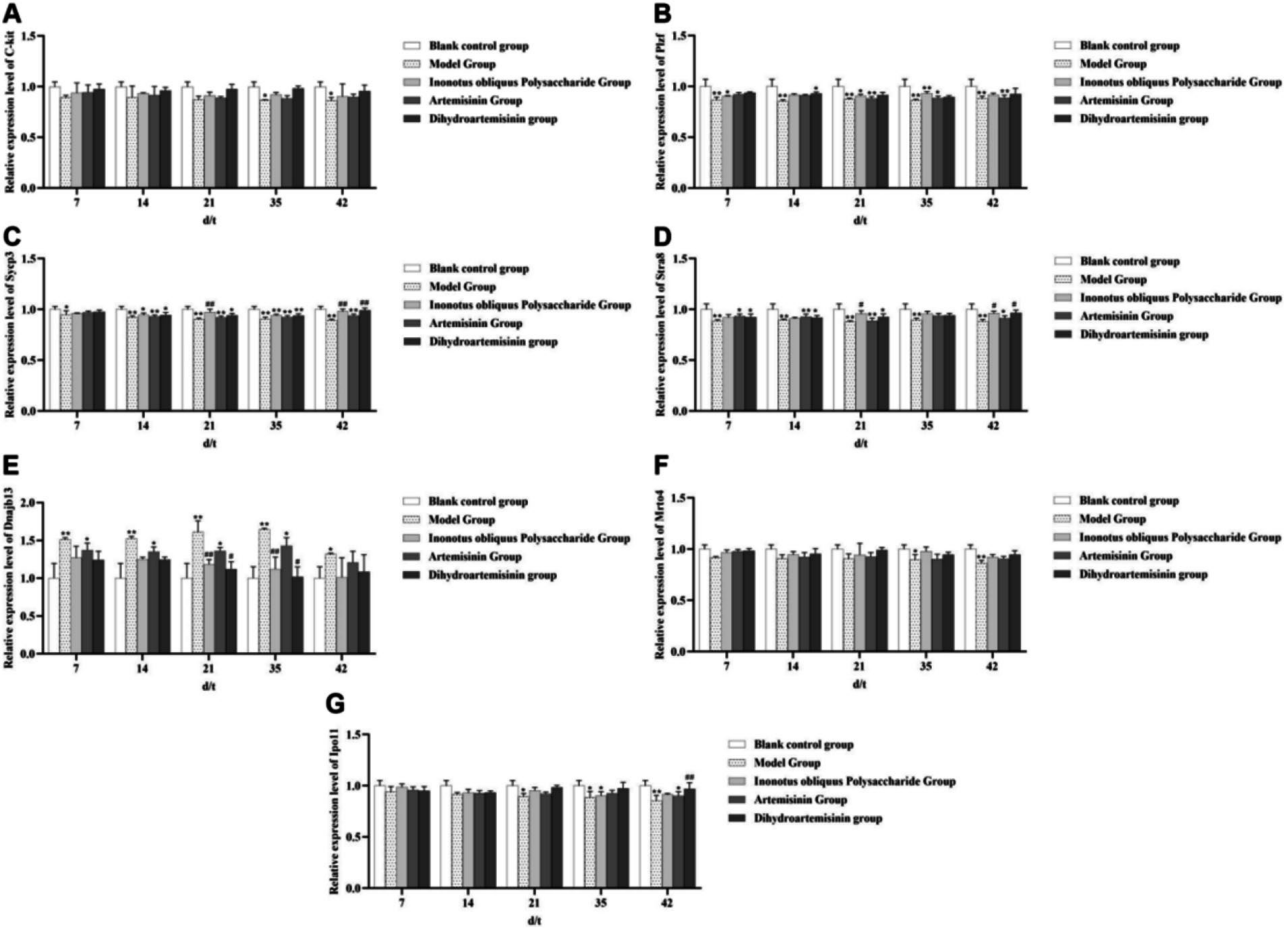
Genetic testing of spermatogenesis. **(A)** Expression level of C-kit gene in mouse testicular tissue. **(B)** Expression level of Plzf gene in mouse testicular tissue. **(C)** Expression level of *Sycp3* gene in mouse testicular tissue. **(D)** Expression level of *Stra8* gene in mouse testicular tissue. **(E)** Expression level of *Dnajb13* gene in mouse testicular tissue. **(F)** Expression level of *Mrto4* gene in mouse testicular tissue. **(G)** Expression level of *Ipo11* gene in mouse testicular tissue. Compared with the blank control group, *p* < 0.01; *Compared with the blank control group, *p* < 0.05. ##Compared with the model group, *p* < 0.01. #Compared with the model group, *p* < 0.05.

The c-kit gene expression level in the infected model group was significantly lower than that in the blank control group from test days 35 to 42 (*p* < 0.05). The infected model group exhibited a significantly lower PLZF gene expression level than the blank control group from test days 7 to 42 (*p* < 0.01). The Mrto4 gene expression level in the model group was significantly lower than that in the blank control group on test days 35 and 42 (*p* < 0.05 and *p* < 0.01, respectively). However, compared to the infected model group, the three treatment groups showed no significant change in the expression levels of c-kit, PLZF, and Mrto4 genes. The infected model group displayed a significantly lower SYCP3 gene expression level than the blank control group on test days 7 to 42 (*p* < 0.05). In contrast, the IOPs-treated group showed a significantly higher SYCP3 gene expression level than the infected model group from treatment days 21 to 42 (*p* < 0.01). On the 42nd test day, the SYCP3 gene expression level was significantly higher in the DHA-treated group than in the infected model group (*p* < 0.01). The Stra8 gene expression level was significantly lower in the infected model group than in the blank control group from test days 7 to 42 (*p* < 0.01). On the 42nd test day, both IOPs-treated and DHA-treated groups showed significantly higher Stra8 gene expression levels than the infected model group (*p* < 0.05). The Dnajb13 gene expression level was significantly higher in the infected model group than in the blank control group on test days 7 to 42 (*p* < 0.05). On test days 21 and 35, both IOPs-treated and DHA-treated groups exhibited significantly lower Dnajb13 gene expression levels than the infected model group (*p* < 0.01 and *p* < 0.05, respectively). On test days 21 and 42, the model group showed significantly lower Iop11 gene expression level than the blank control group (*p* < 0.05 and *p* < 0.01, respectively). Moreover, on test day 42, the Iop11 gene expression level of the DHA-treated group was significantly higher than that of the model group (*p* < 0.01; Figure 14).

## 4 Discussion

### 4.1 General introduction

N. caninum is an obligate, intracellular parasitic protozoan that causes abortion, stillbirth, and reproductive system damage in pregnant animals, resulting in substantial economic losses to animal husbandry (22, 23). Currently, there is no effective vaccine or drug for controlling neosporosis. The present study found that IOPs, ART, and DHA can effectively alleviate the symptoms of reproductive system damage in male mice with N. caninum infection. Some studies have found that DHA is more stable, less toxic, and has a better antimalarial effect compared to ART (9). Other studies have also shown that IOP has a certain protective effect on mice infected with Neospora caninum (24). In this study, all results collectively indicate that DHA is more effective than ART in treating male mice infected with Neospora caninum, but the difference between DHA and IOP is not significant at present. This requires further verification through more *in vivo* experiments, such as studies on male mice and their offspring.

### 4.2 Sperm parameters

The assessment of sperm quality primarily involves the following parameters: sperm viability, teratozoospermia rate (percentage of abnormal forms), sperm concentration, sperm motility, and one-hour survival rate. Sperm quality is considered abnormal and suboptimal if the viability falls below 60%, the teratozoospermia rate exceeds 20%, the concentration is lower than 9.5 × 10^6^/mL, the motility is less than 70%, or the one-hour survival rate is below 60%. Such abnormalities may adversely affect offspring reproduction to varying degrees (25, 26). Therefore, the sperm quality of mice in the infected model group was significantly decreased, which manifested as reduced sperm motility, increased deformity rate, and decreased sperm density; this finding was consistent with the results of previous studies that N. caninum can interfere with male reproductive function (19). Additionally, the sperm quality parameters (sperm motility, deformity rate, and density) of mice in the drug treatment group were significantly improved compared to those in the infected model group (27), with DHA showing the most prominent effect, which may be related to its high-efficiency antiparasitic activity and rapid metabolic characteristics. In this study, on the 35th day of drug administration, a decrease in sperm count was observed in the IOPs-treated groups mice. Combined with subsequent expression experiments of spermatogenesis-related genes, abnormalities in the expression of three genes, namely Plzf, Sycp3, and stra8, were observed on the 35th day of drug administration. However, no existing research can explain why this phenomenon occurs. This may require deeper investigation.

### 4.3 Histopathological changes

The reproductive organs are crucial components of the body’s reproductive system, with the testis and epididymis being two significant internal reproductive organs in male animals. Previous studies have indicated that Neospora can harm the reproductive system of male animals (19). In the HE staining results, it was observed that the testes and epididymis of mice in the blank control group exhibited normal structures. However, in the model group, abnormal arrangement of spermatogenic cells at various stages, shedding of spermatogenic cells, reduction in sperm count, shortening of epithelial cells in the epididymal ducts, and even rupture of the duct walls were evident. Following treatment with polysaccharides from IOPs, ART, and DHA, the pathological changes in the testes and epididymis tissues showed varying degrees of improvement. The number of sperm in the lumens increased, the number of shed spermatogenic cells decreased, the arrangement of spermatogenic cells became clearer, the number of interstitial cells between the ducts increased, and there was a slight rupture of the duct walls. Transmission electron microscopy results revealed normal morphology and structure of various organelles in the tissues and organs of mice in the blank control group. In contrast, the organelles within the cells of mice in the model group exhibited numerous abnormalities, including extensive vacuolization of mitochondria, reduced numbers of Golgi complexes and ribosomes, uneven distribution of peripheral dense fibers in sperm, shrinkage of nuclear membranes, and unclear boundaries. After treatment with polysaccharides from IOPs, the vacuolization of mitochondria significantly improved, the nuclear membrane remained normal, and malformed sperm were rarely observed. Based on the analysis of these three experimental results, it is confirmed that Neospora can cause damage to various tissues and organs, particularly the testes and epididymis, in male mice. However, polysaccharides from IOPs, ART, and DHAn can all mitigate this damage to varying extents, thereby enhancing the body’s resistance to Neospora.

### 4.4 Oxidative stress responses

N. caninum infection can induce oxidative stress, leading to a high MDA level and decreased ACP activity in testicular tissue, which suggests exacerbated lipid peroxidation and impaired Sertoli cell function (28–30). NO is a highly reactive gaseous molecule produced by macrophages and formed in liver cells, duodenal cells, and vascular endothelial cells. Studies abroad have shown that high concentrations of NO can inhibit the secretion of testosterone in the body, thereby suppressing sperm production. AsAb is an antibody produced by the male animal’s body itself. It can hinder the normal sperm production, cause sperm agglutination and immobilization, interfere with sperm energy acquisition, thereby reducing sperm survival rate and vitality, leading to infertility symptoms in male animals (31, 32). The results of this study show that the NO level in the serum of mice in the model group is significantly higher than that in the blank control group. However, after administration of three drugs, namely IOP, ART, and DHA, the NO level in the serum gradually returns to normal with time. Compared to the blank control group, the AsAb concentration in the serum of mice in the model group increases and reaches its peak on day 35 of the experiment, indicating that Neospora can cause testicular tissue damage. Furthermore, IOP, ART, and DHA can improve this result; after administration of these drugs, the AsAb concentration decreases relatively. This suggests that Neospora can increase the concentration of AsAb, thereby hindering normal sperm production, reducing sperm motility and vitality, and causing reproductive disorders in male mice.

### 4.5 Immunomodulation

IgG antibody is considered a key indicator of the humoral immune response triggered by Neospora infection. Neospora infection elicits a Th1-type immune response, and fluctuations in cytokine levels are regarded as a crucial feature of cellular immunity, particularly factors like pro-inflammatory cytokines IL-4 and TNF-*α*, which are regarded as decisive factors in combating Neospora infection. IFN-*γ* is primarily produced by activated Th cells and NK cells, while IL-6 stimulates the growth and activation of B cells, leading to antibody production and inhibiting Th1-type immune responses and IFN-γ production. IgE is associated with type I allergic reactions (33–35). The serum levels of proinflammatory factors (TNF-α, IFN-γ, and IL-6) and immunoglobulins (IgG1, IgG2a, and IgE) increased in the infected model group, while the inflammatory response was significantly attenuated after drug treatment, indicating that these three drugs may inhibit excessive inflammatory response by regulating Th1/Th2 immune balance.

### 4.6 Gene expression alterations

qPCR and FCM analysis demonstrated that N. caninum infection can upregulate the expression of proapoptotic genes (Bax, caspase-3, and p53) and inhibit the expression of antiapoptotic genes (Bcl-2), resulting in excessive apoptosis of spermatogenic cells (36, 37). Drug treatment restored the expression of apoptosis-related genes, with IOPs and DHA showing the optimum effect. Dnajb13 is a member of type II heat shock protein 40 family (Hsp40). In testicular tissue structure, Dnajb13 is localized in the cytoplasm of developing sperm cells and mature sperm tail and plays a key role in normal spermatogenesis. Additionally, Mrto4 is abundantly expressed in testis tissue, and it can regulate polyadenylation and deadenylation of some specific mRNAs during spermatogenesis. Ipo11 influences the expression of TSLC1/IGSF4, which enables spermatogenic cells to develop and mature faster (38). During the spermatogenesis process, the expressed Mrto4 and Ipo11 proteins bind to RNA and regulate this process, which is particularly important for maintaining male reproductive function. The expression of key spermatogenesis-related genes (c-kit, PLZF, SYCP3, and Stra8) and spermatogenesis function-related genes (Mrto4 and Ipo11) was downregulated in the infected model group, and their expression levels were restored following drug treatment; this finding confirmed that these three drugs can improve male reproductive function by regulating spermatogenesis-related pathways.

### 4.7 Summary

The present study systematically evaluated the protective effects of IOPs, ART, and DHA on the reproductive system of male mice with N. caninum infection for the first time and confirmed that these three drugs can improve reproductive function by reducing oxidative damage, regulating immune response, inhibiting apoptosis, and promoting the expression of spermatogenesis-related genes. DHA showed the most significant inhibitory effect among the three drugs, and its administration schedule can be further optimized in the future to enhance its clinical application potential. This study provides an important theoretical basis for developing anti-neosporosis drugs.

## 5 Conclusion

IOPs and DHA remarkably alleviated N. caninum infection-induced damage to the testis and epididymis tissue cells in infected model mice. Both drugs inhibited the apoptosis of testicular spermatogenic cells due to N. caninum infection, regulated the expression of spermatogenesis-related genes, restored the impaired spermatogenic function, and prevented abnormal sperm production. Moreover, IOPs and DHA reduced the number of abnormal sperm in infected model mice; enhanced sperm density and sperm motility; regulated the expression levels of genes associated with antioxidant activity, immune function, testicular cell apoptosis, and spermatogenesis; and improved the immunity level. But ART has no obvious therapeutic effect on male mice infected with Neospora caninum. IOPs and DHA exhibit effective anti-sporozoite activity in male mice, demonstrating their potential as promising candidates for further exploration in the prevention or treatment of sporozoite infections in murine models.

## Data Availability

The datasets presented in this study can be found in online repositories. The names of the repository/repositories and accession number(s) can be found in the article/supplementary material.
